# Machine Learning Helps Identify CHRONO as a Circadian Clock Component

**DOI:** 10.1371/journal.pbio.1001840

**Published:** 2014-04-15

**Authors:** Ron C. Anafi, Yool Lee, Trey K. Sato, Anand Venkataraman, Chidambaram Ramanathan, Ibrahim H. Kavakli, Michael E. Hughes, Julie E. Baggs, Jacqueline Growe, Andrew C. Liu, Junhyong Kim, John B. Hogenesch

**Affiliations:** 1 Division of Sleep Medicine, University of Pennsylvania School of Medicine, Philadelphia, Pennsylvania, United States of America; 2 Center for Sleep and Circadian Neurobiology, University of Pennsylvania School of Medicine, Philadelphia, Pennsylvania, United States of America; 3 Department of Pharmacology and the Institute for Translational Medicine and Therapeutics, University of Pennsylvania School of Medicine, Philadelphia, Pennsylvania, United States of America; 4 Department of Biological Sciences, University of Memphis, Memphis, Tennessee, United States of America; 5 Department of Chemical and Biological Engineering, Koc University, Istanbul, Turkey; 6 Department of Biology, University of Missouri–St. Louis, St. Louis, Missouri, United States of America; 7 Department of Pharmacology, Morehouse School of Medicine, Atlanta, Georgia, United States of America; 8 Department of Biology, University of Pennsylvania, Philadelphia, Pennsylvania, United States of America; University of Geneva, Switzerland

## Abstract

Over the last decades, researchers have characterized a set of “clock genes” that drive daily rhythms in physiology and behavior. This arduous work has yielded results with far-reaching consequences in metabolic, psychiatric, and neoplastic disorders. Recent attempts to expand our understanding of circadian regulation have moved beyond the mutagenesis screens that identified the first clock components, employing higher throughput genomic and proteomic techniques. In order to further accelerate clock gene discovery, we utilized a computer-assisted approach to identify and prioritize candidate clock components. We used a simple form of probabilistic machine learning to integrate biologically relevant, genome-scale data and ranked genes on their similarity to known clock components. We then used a secondary experimental screen to characterize the top candidates. We found that several physically interact with known clock components in a mammalian two-hybrid screen and modulate *in vitro* cellular rhythms in an immortalized mouse fibroblast line (NIH 3T3). One candidate, Gene Model 129, interacts with BMAL1 and functionally represses the key driver of molecular rhythms, the BMAL1/CLOCK transcriptional complex. Given these results, we have renamed the gene CHRONO (computationally highlighted repressor of the network oscillator). Bi-molecular fluorescence complementation and co-immunoprecipitation demonstrate that CHRONO represses by abrogating the binding of BMAL1 to its transcriptional co-activator CBP. Most importantly, CHRONO knockout mice display a prolonged free-running circadian period similar to, or more drastic than, six other clock components. We conclude that CHRONO is a functional clock component providing a new layer of control on circadian molecular dynamics.

## Abstract 2

Two independent studies, one of them using a computational approach, identified CHRONO, a gene shown to modulate the activity of circadian transcription factors and alter circadian behavior in mice.

## Author Summary

Daily rhythms are ever-present in the living world, driving the sleep–wake cycle and many other physiological changes. In the last two decades, several labs have identified “clock genes” that interact to generate underlying molecular oscillations. However, many aspects of circadian molecular physiology remain unexplained. Here, we used a simple “machine learning” approach to identify new clock genes by searching the genome for candidate genes that share clock-like features such as cycling, broad-based tissue RNA expression, *in vitro* circadian activity, genetic interactions, and homology across species. Genes were ranked by their similarity to known clock components and the candidates were screened and validated for evidence of clock function *in vitro*. One candidate, which we renamed CHRONO (Gm129), interacted with the master regulator of the clock, BMAL1, disrupting its transcriptional activity. We found that *Chrono* knockout mice had prolonged locomotor activity rhythms, getting up progressively later each day. Our experiments demonstrated that CHRONO interferes with the ability of BMAL1 to recruit CBP, a bona fide histone acetylase and key transcriptional coactivator of the circadian clock.

## Introduction

Circadian rhythms are ubiquitous in daily life, coordinating the sleep–wake cycle along with oscillations in hormone secretion, blood pressure, and cognitive function [1],[2]. While a central master-pacemaker is located in the suprachiasmatic nuclei (SCN) of the hypothalamus, cell autonomous rhythms are generated throughout the body. The CLOCK/BMAL1 transcriptional complex lies at the core of the molecular clock. These proteins bind E-box elements in the promoters of target genes [3]. The Period and Cryptochrome gene families are prominent among these targets, and their products ultimately repress CLOCK/BMAL1 activity and their own transcription [4],[5]. A second loop regulates *Bmal1* expression through the opposing actions of the REV–ERB and ROR nuclear receptor protein families [6],[7]. Circadian oscillations are in turn subject to multiple layers of control. The casein kinase I proteins (CSNK1D and CSNK1E) and the F-box and leucine-rich repeat proteins (FBXL3, FBXL21) [8]–[10] regulate the nuclear accumulation and/or stability of clock components, respectively. Moreover, recent evidence highlights the importance of metabolic cofactors and histone modifiers (e.g., HDAC3, P300, CBP, SIRT1, and NAMPT) in modulating these feedback loops.

The understanding of circadian timekeeping has demonstrated far-reaching importance. Allelic variation in clock components has been associated with circadian, sleep, and mood disorders [8],[11]–[13]. Mutational and epidemiologic studies have linked clock genes with neoplastic and metabolic phenotypes [2],[14]. However, the current model of the circadian pacemaker is likely incomplete. Indeed, quantitative circadian trait analysis maps most loci to regions unassociated with known clock genes [15]. In an attempt to identify these missing regulatory components, researchers have moved beyond the costly and laborious mutagenesis screens that identified the first clock components [16],[17]. Recent studies have turned to higher throughput genomic and proteomic approaches. A screen for activators of BMAL1 transcription [7], a screen for proteins that bind CLOCK [18], and proteomic analysis of the BMAL1 [19] and PERIOD [20],[21] protein complexes have all identified proteins that function in circadian control.

Here we present an alternative, computer-assisted approach aimed at accelerating clock gene discovery. We used probabilistic machine learning to integrate heterogeneous, genome-scale datasets [22]–[24] and identify candidate clock genes that functionally resemble known clock components. We screened the top candidates for physical interactions with a subset of clock components using a mammalian two-hybrid assay. Candidates were further screened for circadian function in an *in vitro* system. We focused our attention on three promising initial candidates. Here we demonstrate the utility of this approach with data from the first of these candidates, *Gene Model 129* (*Gm129*), to have its circadian function characterized in both cells and knockout mice. We confirmed that *Gm129* physically interacts with core clock genes and regulates the molecular oscillator. In addition, *Gm129* oscillates in multiple tissues, functionally represses the activity of the CLOCK/BMAL1 transcriptional complex, and most importantly, influences the free-running circadian period of locomotor activity in mice. In view of its role as a *c*omputationally *h*ighlighted *r*epressor *o*f the *n*etwork *o*scillator, we have renamed the gene *Chrono*.

## Results and Discussion

In order to identify novel “core clock genes,” we considered physiologically relevant features that define core circadian components: (1) Core clock components cycle with a ∼24-h period. (2) Core clock gene mutation or knockdown affects circadian behavioral rhythms. (3) Core clock genes interact with other core clock genes. (4) Core clock genes are expressed in most tissues. (5) Core clock genes are phylogenically conserved between vertebrates and flies.

**Figure 1:**
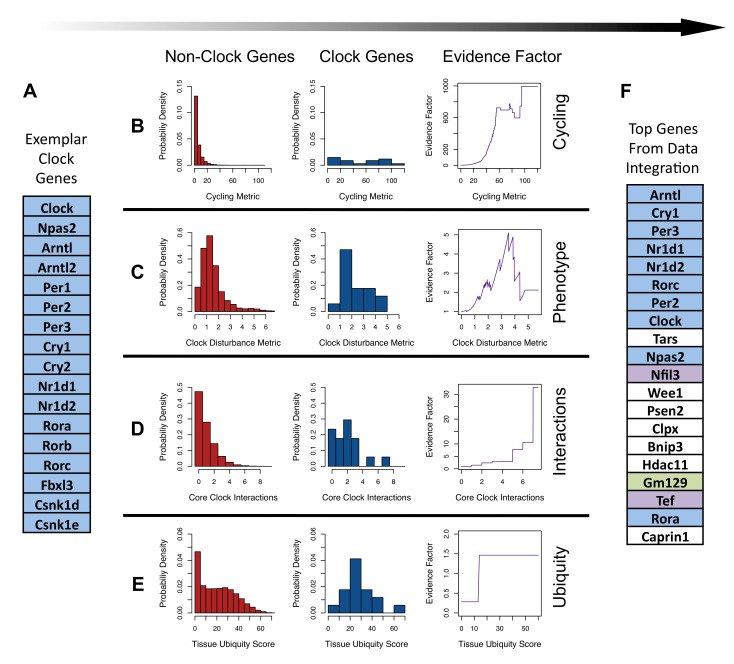
Integration of core clock features. (A) List of exemplar core clock genes used as example models of core clock components. (B–E) Metric functions describing core clock features were generated from published data. Distributions of these metrics among nonclock genes (left panel) and exemplar clock genes (center panel) were used to construct evidence factors (right panel). (B) Cycling was evaluated using time-course microarray data from liver, pituitary, and NIH 3T3 cells. (C) Circadian disturbance metric quantifies the influence of RNAi-mediated gene knockdown on circadian dynamics in the U2OS model system. (D) The interaction metric counts the number of interactions inferred between each gene and the exemplar set of core clock genes. (E) The tissue ubiquity scores were taken from an EST database. (F) List of 20 genes most likely to have a core circadian function as determined by evidence factor integration. Genes highlighted in blue were included in the exemplar training set. Genes highlighted in purple were not in the training set but have been identified as having a role in the circadian clock. *Gm129* was selected for further characterization.

Importantly, as is demonstrated by our exemplar set of known clock genes (Figure 1A), none of these features are absolute requirements: The canonical circadian gene *Clock*, for example, does not cycle robustly in the pituitary [25] or SCN [26]. Individual knockdown of either the *Nr1d1* or *Nr1d2* genes has minimal phenotypic effect [27]. Rather, these features lie on a continuum, each lending some support to a given gene having a core circadian function.

We used published, genome-wide datasets that provide information on each of these features and developed simple, albeit imperfect, metrics to quantify each feature. These metrics were designed to reward clock-like features.

### Core Clock Metrics

#### Cycling

In order to assess transcript cycling, we reanalyzed high-resolution time course microarray data for liver, pituitary, and NIH 3T3 cells [25]. As detailed in the Materials and Methods section, we combined the *p* values obtained by evaluating cycling in each tissue to create a single general cycling metric (M_Cyc_) for each gene. Higher values of M_Cyc_ correspond to more robust cycling in this combination of tissues. Compared to nonclock genes (Figure 1B, Left), the distribution of M_Cyc_ among the exemplar clock genes (Figure 1B, Center) is shifted far to the right with clock components demonstrating more robust cycling. Intuitively, a very high value of M_Cyc_ provides some suggestion that a gene may belong to the set of core clock genes.

#### Phenotype

We used data from a genome-wide RNA interference (RNAi) screen identifying *in vitro* circadian modulators [28] to generate a circadian disturbance metric (M_Dist_). By construction, larger values of M_Dist_ reflect greater influence on *in vitro* rhythms (Figure 1C). In comparison to nonclock genes, the distribution of M_Dist_ among core-clock genes is shifted to the right with clock genes demonstrating more impact on cellular circadian phenotypes. Interestingly, the most extreme values in M_Dist_ did not result from the knockdown of known clock genes. A second, small mode of extreme M_Dist_ values was observed in the screen and may have resulted from knockdowns that nonspecifically affected cellular health [28].

#### Network interactions

A genome-wide database of functional genetic interactions inferred from radiation hybrid mapping was used to count the number of connections between each gene and the exemplar set of clock components (M_int_) [29]. The distributions of M_int_ within the genome at large and among the exemplar set of core clock components are shown in Figure 1D. Clock genes form a tightly connected network with core clock genes being more likely to have functional connections to other clock genes.

#### Ubiquity

We counted the number of tissues in which each gene has been definitively identified via Expressed Sequence Tags (ESTs) [30]. The plurality of nonclock transcripts are detected in only 1–2 tissues, and less than half of all transcripts have been found in 15 or more murine tissues. In comparison, the exemplar core clock genes are more widely expressed (Figure 1E).

#### Phylogenic conservation

For each included gene, we utilized the Homologene database [31] to determine if an annotated *Drosophila melanogaster* homologue has been identified. While this feature was included in the final model, there was only a small difference between the fraction of clock genes possessing *Drosophila* homologues and the fraction of nonclock genes possessing such homologues. The modest fraction of exemplar genes with annotated homologues and this small difference likely reflects the strict criteria used in constructing the Homologene database and may underestimate the value of this feature in the ultimate weighting.

### Creation of Circadian Evidence Factors

Using the above empirical distributions and a modified version of the Naïve Bayes learning algorithm, we quantified the evidence provided by each feature that a given gene is a member of the core circadian network [22],[32]. We relied on the prior assumption, informed by experimenter judgment, that increasing possession of each of these features lends increasing evidence of a role in the circadian clock. We used the empirical cumulative distribution function (ECDF) describing exemplar “clock genes” or “nonclock genes” to estimate the probabilities that a randomly selected “clock gene” or “nonclock gene” would possess a metric value at least as extreme as the one observed. We term the ratio of these probabilities a “circadian evidence factor” (Materials and Methods, Eq. 4). The evidence factors arising from particular features and metric value are shown in the right panels of Figure 1B–E).

The evidence amassed from all five features is encapsulated by a “combined evidence factor.” Computation of combined evidence factors requires knowledge of the joint cumulative probability distributions for these features among both “clock genes” and “nonclock genes.” These joint cumulative distribution functions are “learned” from the examples under the “Naïve” assumption of conditional independence. Evidence factors from each individual feature are multiplied to calculate the combined evidence factor (Materials and Methods, Eq. 6). This approach differs from the standard Naïve Bayes statistical learning approach only in that cumulative distribution functions are used rather than probability density functions.

We ranked genes based on this combined evidence. The top 20 candidates (Figure 1F) include 10 of the exemplar clock components along with *Tef* [33] and *Nfil3* [34], two genes with established circadian functions. Moreover, *Wee1*, a canonical cell cycle gene, is known to be regulated by the circadian clock [35]. Although, to our knowledge, the hypothesis that *Wee1* directly regulates clock function has not been tested. Inspecting the top 50 ranked genes, several other genes known to be involved in the circadian clockworks appear. These include *Dbp* [36], *Insig2* [37], and *Nampt* [38].

### Evidence Factors Predict Circadian Function

In order to evaluate the utility of this ranking in the discovery of novel clock genes, we applied 10-fold cross-validation. We sequentially removed all possible pairs of clock components from the exemplar distribution, ignoring our prior knowledge of their role in orchestrating circadian rhythms. In each case, we then recomputed the combined evidence factors based on this reduced knowledgebase and tested our ability to “rediscover” these clock genes using different ranking cutoffs. Based on this analysis, we estimate that ∼50% of true clock components would be recovered by screening the top 50 genes (Figure S1A). We also compared the use of evidence factors with two prepackaged machine learning algorithms. Using the same features, we ranked genes using a Gaussian Naïve Bayes classifier and a Flexible Naïve Bayes classifier [39]. The three methods all yield comparable performances using cutoffs less than ∼1,000, but the evidence factor method outperforms the other two beyond this point. Importantly, the top candidates from all three methods show a very high degree of overlap (Figure S1B).

Only rankings from the evidence factor approach were used in selecting genes for further screening. However, results from all three probabilistic learning methods are presented in the Supporting Information section. The cycling feature makes the largest single contribution to the combined evidence factors, but it does not completely dominate this ranking. Hundreds of genes demonstrate strong cycling in the tissues analyzed and other features determine the relative ranking among these. Moreover, some candidates, like *Hdac11*, are largely prioritized based on the combined strength of other features.

Given the rarity of bona fide clock genes, any method that is not 100% specific will result in a number of false positives. As the ranking cutoff is increased, the number of nonclock genes incorrectly identified will also increase. As in other screening applications where one is searching for a “needle in a haystack,” a secondary validation of candidates is needed. Assuming different numbers for the total number of core clock components, we estimated the false positive rate for different screening cutoffs (Figure S1C).

The ultimate value of this approach will be determined by its ability to identify previously unrecognized clock components. We tested the top 25 novel candidates for physical interactions with a subset of proteins from the negative arm of the molecular clock (BMAL1, BMAL2, CLOCK, NPAS2, CRY1, CRY2. PER1, PER2, and PER3). Three of these candidates (*Gm129*, *Ifitm1*, and *Cbs*) demonstrated both physical binding with at least one of the included clock components and a statistically significant change in circadian reporter period after knockdown in the NIH 3T3 model system (Figure S2). Of note, although *Gm129* might have been identified simply by its strong cycling, *Cbs* and *Ifitm1* are identified by virtue of a combination of features. Bellow we present a more detailed investigation of the previously uncharacterized candidate, *Gm129*, here renamed *Chrono*. These data show that *Chrono* meets the formal definition of a mammalian circadian clock gene.

### Chrono mRNA Cycles in Multiple Tissues

**Figure 2:**
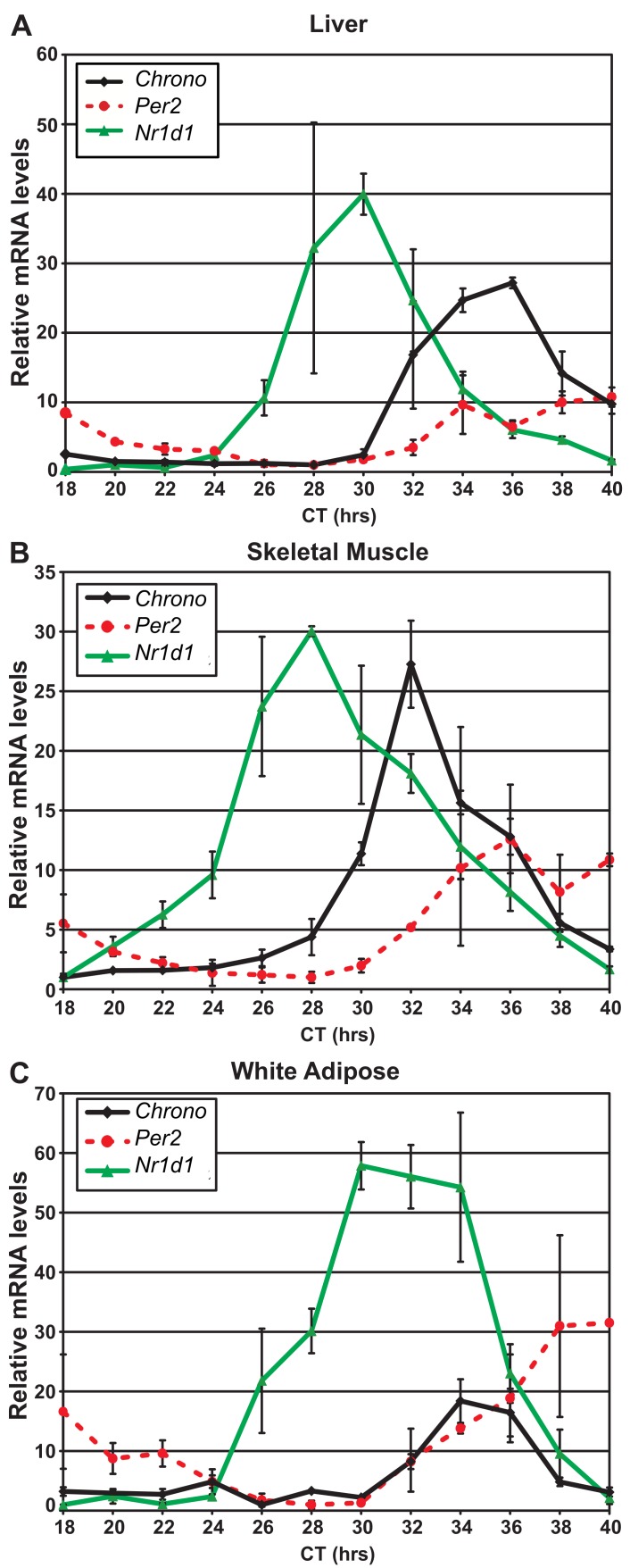
*Chrono* transcript demonstrates circadian oscillations in peripheral tissues. qPCR was used to measure transcript abundance of *Chrono*, *Per2*, and *Nr1d1* in (A) liver, (B) skeletal muscle, and (C) adipose tissue. Circadian variation is observed in each tissue with the amplitude of *Chrono* oscillations comparable to that of *Per2* and *Nr1d1*. Data shown are the average of 3–4 biological replicates.

Our previous microarray data suggested that *Chrono* expression cycles with a 24-h period in liver, pituitary, and NIH 3T3 cells [25],[40]. We used quantitative PCR (qPCR) to confirm cycling in the liver and further evaluated transcript cycling in skeletal muscle and white fat (Figure 2). The circadian oscillations in *Chrono* expression are of a similar magnitude to those observed for known clock factors *Nr1d1* and *Per2*. Consistent with our results, temporal profiling in rat skeletal muscle [41] and lung [42], as well as mouse SCN [43], also revealed daily oscillations in *Chrono* expression. Several genome-wide, ChIP-seq studies in mouse liver [43]–[45] have identified the E-boxes in the *Chrono* gene promoter among those genomic regions most tightly bound by BMAL1 protein. Time course microarray studies from SCN and liver demonstrate that *Chrono* expression is reduced in *Clock* mutant animals and loses circadian rhythmicity (Figure S3A) [46],[47]. Moreover, *Chrono* expression becomes arrhythmic in the livers of *Cry1/Cry2* double knockout animals (Figure S3B) [48]. In total, *Chrono* demonstrates robust circadian expression in multiple tissues and appears to be directly regulated by the molecular clock.

### Chrono Physically and Functionally Interacts with the Circadian Clock

**Figure 3:**
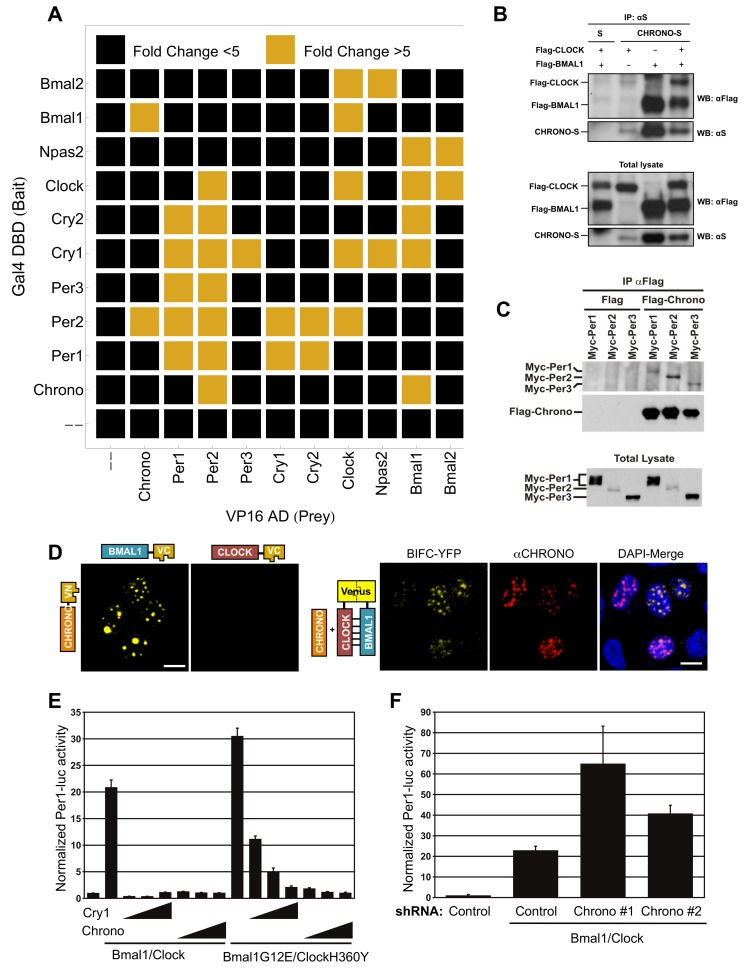
Physical and functional interactions of CHRONO. (A) Results from a matrix of mammalian two-hybrid assays between known circadian clock components fused to Gal4 DNA binding domain (Gal4 DBD) or VP16 activation domain (VP16 AD). Black and gold indicate bait–prey interactions that resulted in less or greater than 5-fold activation of the 4XUAS reporter, respectively. Co-IP with tagged CHRONO confirms complex formation with (B) BMAL1 and (C) PER2. (D) C- and N-terminal regions of Venus, an enhanced florescent protein, were fused with identified constructs. A yellow bi-molecular fluorescence signal identifies interactions. (E) HEK 293T cells were transiently transfected with a Per1:luc reporter, wild-type, or mutant *Bmal1/Clock*, and increasing amounts of *Cry1* or *Chrono*. BMAL1/CLOCK point mutants are resistant to CRY1-mediated repression but sensitive to CHRONO. (F) The ability of native CHRONO to repress BMAL1/CLOCK activity was determined by transient transfection with two distinct shRNA constructs directed against *Chrono*. The indicated plasmids were co-transfected with the Per1-luc reporter into HEK 293T cells. Average activities and standard deviations from reporter assays were determined from independent biological triplicates.

We employed a mammalian two-hybrid screen to identify physical interactions between CHRONO and a subset of known clock components. As expected, many core clock proteins physically interacted, as indicated by specific activation of a UAS:Luc reporter in transfected Human Embryonic Kidney 293 cells containing the SV40 T-Antigen (HEK 293T) (Figure 3A, Table S1). Interactions between CHRONO and both BMAL1 and PER2 were also observed, with >20-fold induction of luciferase activity. BMAL1–CHRONO and PER2–CHRONO complex formation were confirmed through co-immunoprecipitation (co-IP) (Figure 3B and C). Bi-molecular Fluorescence Complementation (BiFC) using Venus, an enhanced yellow fluorescent protein (YFP), was then used to map BMAL1/CHRONO interactions to cell nuclei (Figure 3D). Notably, when S-tagged CHRONO was overexpressed with both BMAL1 and CLOCK BiFC fusion proteins, CHRONO appeared to colocalize with the CLOCK/BMAL1 heterodimer in nuclear bodies, suggesting that CHRONO continues to interact with BMAL1 while part of this functional circadian complex.

To evaluate the functional consequences of these physical interactions, we monitored *Per1*:luciferase activity in unsynchronized HEK 293T cells transiently transfected with *Clock/Bmal1*. *Per1*:luc reporter activity is enhanced by *Clock/Bmal1* transfection but repressed by the overexpression of either *Cry1* or *Chrono* (Figure 3E). As has been previously demonstrated, CLOCKH360Y and BMAL1G612E missense mutants are resistant to CRY-mediated repression [49]. In contrast, CHRONO-mediated repression is unaffected by these point mutations (Figure 3E). The same pattern was observed in the expression of *Nr1d1*, an endogenous CLOCK/BMAL1 target (Figure S4). Alternatively, CHRONO knockdown augments *Per1*:luc reporter activity (Figure 3F).These data suggest that CHRONO and CRY1 have distinct binding sites and/or functional mechanisms.

### Endogenous Chrono Expression Modulates *in Vivo* Circadian Oscillations

**Figure 4:**
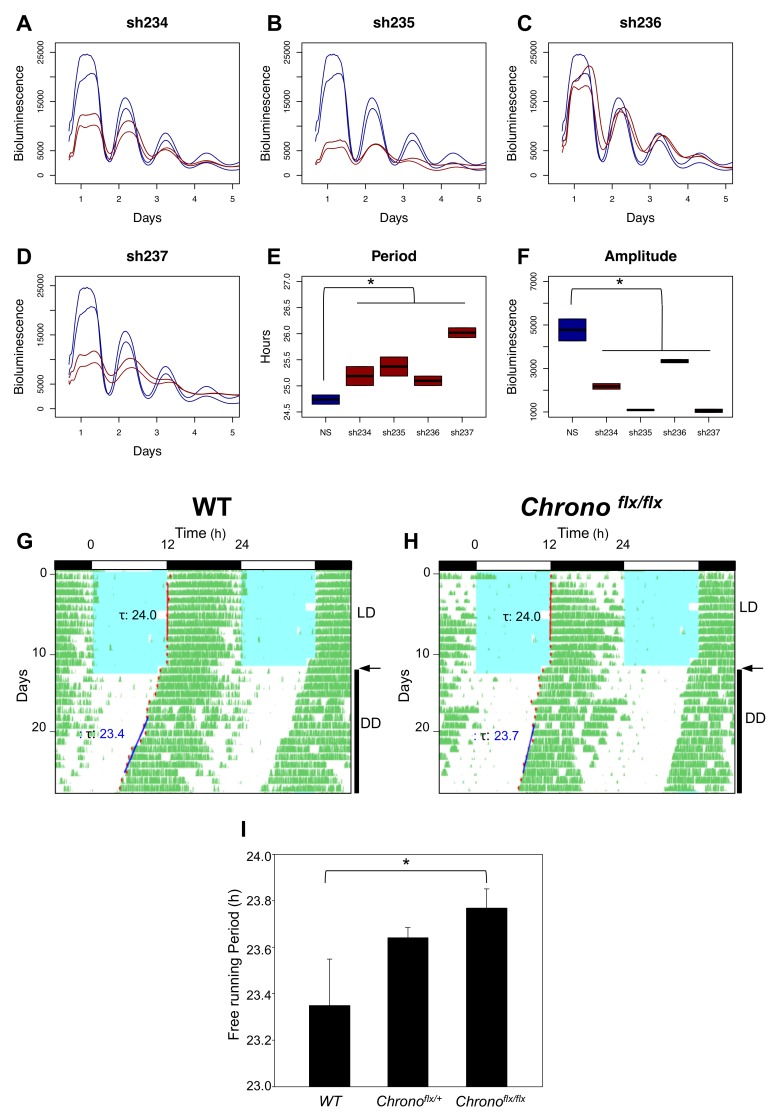
Influence of CHRONO on *in vitro* and *in vivo* rhythms. (A–D) Raw bioluminescence data from NIH 3T3 fibroblasts expressing BMAL:dLUC reporter are plotted after transfection with four shRNA constructs targeted against *Chrono*. Control and *Chrono* knockdown tracings are depicted in blue and red, respectively. Two replicates are shown. The period (E) and amplitude (F) of the observed rhythms are plotted. Representative wheel-running activity records for (G) wild-type control and (H) *Chrono^flx/flx^* knockout mice. Blue shading indicates light exposure during the initial 12∶12 h, L∶D cycle. Arrows indicate transition to constant darkness. Regression lines fit to activity onset and computed period are shown. (I) Periodogram estimates of observed periods from wild-type (*n* = 5), *Chrono^flx/+^* (*n* = 8), and *Chrono^flx/flx^* mice (*n* = 6). Error bars indicate standard error of the mean.

Small interfering RNA (siRNA) mediated knockdown of *C1orf51*, the human homologue of *Chrono*, markedly dampened circadian oscillations in a genome-wide circadian screen [28]. Using NIH 3T3 cells expressing a *Bmal1*:dLuc reporter as a second model system, we tested the effects of four different short hairpin RNA (shRNA) constructs that reduced *Chrono* transcript expression and protein abundance (Figure S5). Comparing the pooled results to control demonstrates that *Chrono* knockdown reduces amplitude and increases circadian period (Figure 4A–F). To definitively establish the role of CHRONO in modulating circadian behavior, we obtained transgenic mice from the Knockout Mouse Project [50]. These mice incorporate a transgenic construct (Figure S6A) whereby the *Chrono* encoding region is flanked by Lox-P sites (*Chrono^flx/flx^*) and utilizes a “knockout-first” cassette [51]. The transgenic allele is a knockout at the level of RNA processing. We mated heterozygous transgenic mice to obtain homozygous *Chrono* knockout mice (*Chrono^flx/flx^*), wild-type littermate controls (*Chrono^+/+^*), and heterozygotes (*Chrono^flx/+^*). qPCR confirmed that, when compared to wild-type littermate controls, mRNA expression was halved in heterozygotes (*Chrono^flx/+^*) and abolished to basal levels in homozygote knockouts (*Chrono^flx/flx^*) (Figure S6B and C). As shown in Figure 4G and H, wild-type, heterozygous, and homozygous knockouts were all well entrained to the 12∶12 light∶dark (L∶D) cycle and maintained a 24-h period. Under free-running conditions, homozygous *Chrono* knockouts exhibited a statistically significant (*p*<0.05) ∼25-min increase in circadian period as compared to wild-type controls (Figure 4I). Heterozygous knockouts display an intermediate period. The magnitude of this period change is similar to that observed in *Clock* (∼20 min) [52], *Per1* (∼40 min) [53], *Per3* (∼30 min) [54], *Nr1d1* (∼20 min) [6], *Rorb* (∼25 min) [55], and *Npas2* (∼12 min) [56] knockout animals. These data strongly suggest that endogenous *Chrono* expression plays an important regulatory role in the mammalian circadian clock.

Light, however, does not appear to directly influence CHRONO expression in the SCN. The SCN microarray data of Jagannath et al. does not reveal a significant change in *Chrono* expression following a nocturnal light pulse [57]. Moreover, in our own experiments, the phase shifting response of *Chrono* knockout mice to light pulses at ZT16 or ZT22 are not significantly different from control. Thus the primary role of CHRONO in the circadian clock appears to be in modulating core oscillator function and output timing rather than oscillator entrainment.

### CHRONO Binds the C-Terminal Region of BMAL1

**Figure 5:**
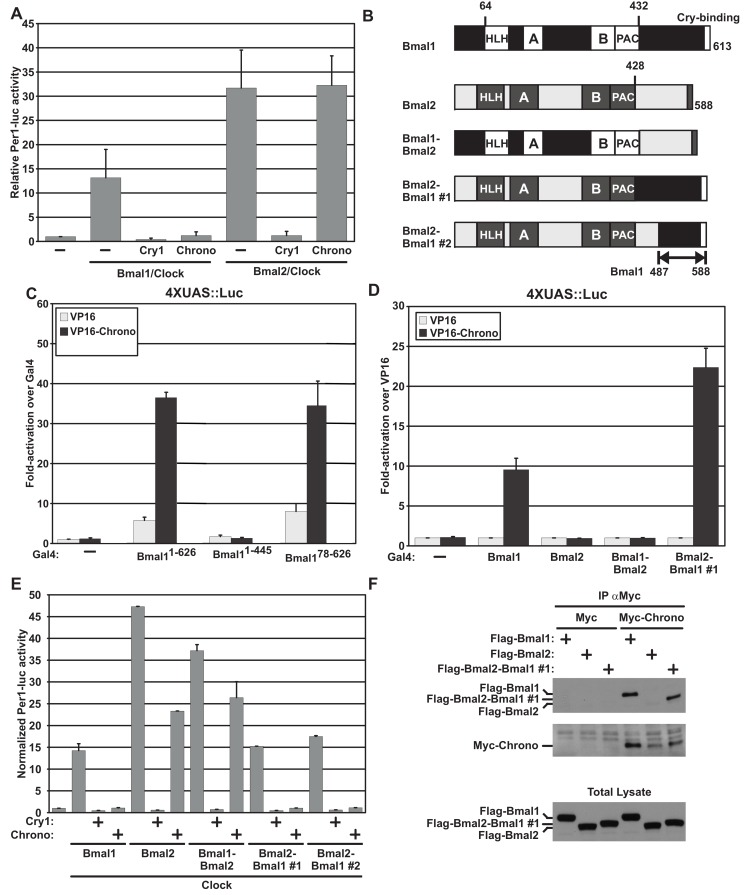
CHRONO interacts with the C-terminus of BMAL1 but not BMAL2. (A) Overexpression of either BMAL1 or BMAL2, along with CLOCK, activates *Per1*:Luciferase reporter activity. Both are repressed by overexpression of CRY1. CHRONO specifically represses BMAL1-induced reporter activity. (B) BMAL1 and BMAL2 have similar structures with conserved bHLH DNA binding domains and PAS A and B interaction domains. BMAL1 contains a unique C-terminal region. Chimeric proteins were constructed by swapping corresponding domains from each protein as shown. Two-hybrid screening in HEK 293T cells demonstrates that BMAL1 truncation mutants (C) and chimeric proteins (D) that contain the 487–586 region of BMAL1 bind CHRONO and induce UAS:Luc reporter expression. This region is adjacent to but distinct from the annotated CRY1 binding site. (E) All BMAL1–BMAL2 constructs induce *Per1*-luc reporter activity in HEK 293T cells. In all constructs, reporter signal is repressed by the addition of CRY1. Functional repression by CHRONO is limited to BMAL constructs containing the implicated binding domain. (F) In cells overexpressing MYC–CHRONO along with BMAL1, BMAL2, or a chimeric BMAL2–BMAL1 construct, co-IP confirms complex formation between CHRONO and proteins containing the implicated BMAL1 C-terminal region.

In a recent report, BMAL2 was shown to function as a tissue-specific paralogue of BMAL1 [58]. However, CHRONO specifically binds BMAL1 and not BMAL2 (Figure 3A). Moreover, CHRONO functionally represses the transcriptional activity of the BMAL1/CLOCK complex but not the activity of the BMAL2/CLOCK complex (Figure 5A).

In order to identify the region of BMAL1 required for CHRONO binding, we generated mutant BMAL1 proteins with truncated N- or C-terminal regions (BMAL1^78–626^, BMAL1^1–445^) (Figure 5B) and tested their interaction with CHRONO using the mammalian two-hybrid assay. Deletion of the C-terminal domain of BMAL1 (BMAL1^1–445^) completely abolished CHRONO binding, whereas deletion of the N-terminal domain (BMAL1^78–626^) had no effect (Figure 5C). We next exploited the strong sequence homology between BMAL1 and BMAL2 to localize the CHRONO binding site within the BMAL1 C-terminal region. We swapped corresponding sections of the BMAL1 and BMAL2 C-terminal domains. As expected, the construct containing the N-terminal of BMAL1 and the full C-terminal of BMAL2 (BMAL1–BMAL2) did not interact with CHRONO in the two-hybrid assay and was relatively immune to CHRONO-mediated repression (Figures 5C–E). A chimeric protein including the N-terminal region of BMAL2 with the longer BMAL1 C-terminus (BMAL2–BMAL1#1) interacted with CHRONO and phenocopied wild-type BMAL1 with regard to CHRONO-mediated repression (Figure 5D–F). Sequence alignment between C-terminal domains of BMAL1 and BMAL2 reveals a region of poor alignment (514–594). Insertion of this unique region of the BMAL1 protein (514–594) into BMAL2 C-terminus rendered the chimeric protein (Bmal2–Bmal1#2) responsive to CHRONO-induced repression (Figure 5E–F). This CHRONO binding region is adjacent to, but distinct from, the CRY1 interacting terminus [59]. Thus, CHRONO functions as a specific transcriptional co-repressor of BMAL1 through interaction with a unique C-terminal domain adjacent to the CRY1 binding region. This domain is both necessary and sufficient for physical and functional interactions with CHRONO.

### CHRONO Abrogates CBP/BMAL1 Binding

**Figure 6:**
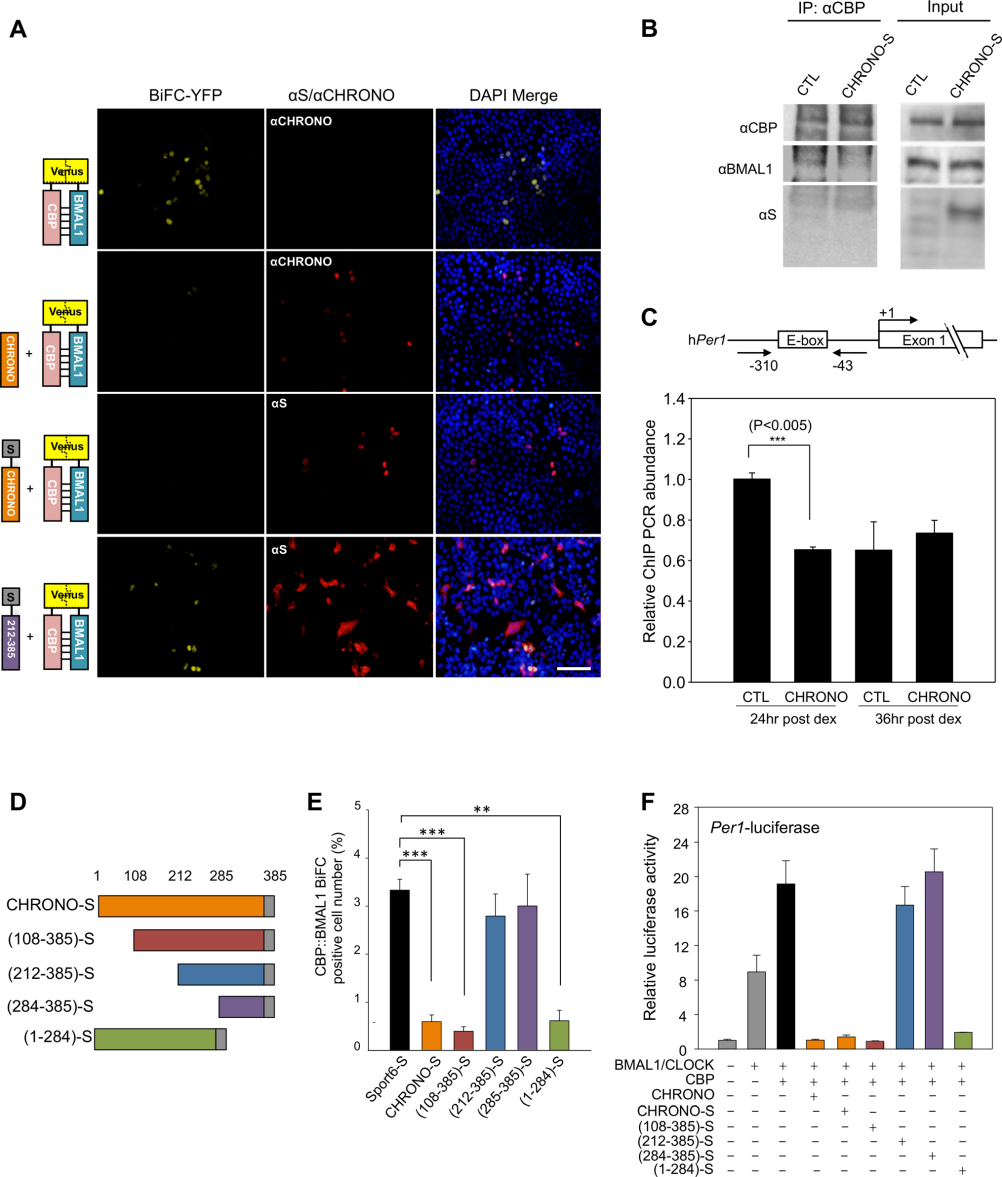
CHRONO interferes with BMAL1–CBP binding. (A) BiFC was used to observe BMAL1–CBP interactions in the nuclei of HEK 293T cells. Co-expression of intact or S-tagged CHRONO reduced the complementation signal. Expression of the 212–385 CHRONO truncation mutant had no discernable effect. (B) IP confirms CHRONO-mediated interference in BMAL1/CBP complex formation. Endogenous protein was immunoprecipitated with anti-CBP antibody followed by immunoblotting as indicated. (C) ChIP qPCR analysis was used to evaluate the effect of CHRONO on the acetylation of histone H3–K9 near the *Per1* promoter E-box region. Schematic diagram of the human *Per1* promoter and primers used for ChIP assay are shown. Lysates obtained from control U2OS cells and those stably expressing CHRONO were collected 24 and 36 h after dexamethasone synchronization. ChIP DNA samples were quantified by quantitative real-time RT-PCR. Data are mean ± standard error of biological triplicates. (D) Various S-tagged, N-, and C-terminal CHRONO truncation mutants were generated. (E) Percent of cell nuclei demonstrating complementation after overexpression of various CHRONO constructs. (F) Per1:luciferase reporter signal in unsynchronized cells overexpressing BMAL1/CLOCK is enhanced by the transient overexpression of CBP. The effect of the overexpression of CHRONO constructs on reporter activity is shown.

Previous studies suggested that CBP also binds to the BMAL1 C-terminus [59],[60]. Thus, we hypothesized that CHRONO might interfere with BMAL1–CBP binding. We generated plasmids encoding BMAL1 and CBP fused to the C- and N-terminal regions of the Venus YFP. We then utilized BiFC to visualize BMAL1–CBP interactions in HEK 293T cell nuclei. BMAL1–CBP complex formation induced a yellow BiFC signal (Figure 6A). Co-expression of native or S-tagged CHRONO severely dampened BMAL1–CBP complementation. Western blotting (Figure S7A) confirmed stable abundance of BMAL1 and CBP proteins, implicating altered binding as the source of the reduced BiFC signal. Lastly, the ability of CHRONO to interfere with BMAL1–CBP binding was verified by co-IP analysis showing that overexpression of intact CHRONO reduced BMAL1–CBP complex formation (Figure 6B).

A functional impairment in the ability of BMAL1 to recruit CBP is expected to reduce histone acetylation of CLOCK/BMAL1 target regions. To assess the influence of CHRONO on the histone acetyl-transferase activity of the BMAL1/CLOCK complex, we performed a ChIP study using an antibody targeting acetylated histone H3 lysine 9 (H3–K9) (Figure 6C). PCR was used to specifically evaluate H3–K9 acetylation near the *Per1* promoter E-box. Control samples obtained from immortalized human osteosarcoma (U2OS) cells 24 and 36 h after dexamethasone synchronization demonstrated a temporal variation in target acetylation. U2OS cells overexpressing CHRONO demonstrated a blunted temporal profile in *Per1* promoter H3–K9 acetylation, with loss of the increased acetylation normally observed 24 h after synchronization [61],[62].

In order to confirm that abrogated CBP/BMAL1 binding contributes to the CHRONO-mediated modulation of circadian dynamics, we constructed several CHRONO truncation mutants. All constructs that retained the 108–212 region reduced BMAL1–CBP binding as assessed by BiFC (Figure 6D and E). As has been previously demonstrated, overexpression of CBP, along with BMAL1 and CLOCK, enhances *Per1*:luc expression in unsynchronized cells (Figure 6F). Those same CHRONO constructs that abrogated BMAL1–CBP complex formation also repressed CBP-enhanced *Per1*:luc reporter activity (Figures 6E and F and S7B). This pattern of activity among CHRONO truncation mutants was further mirrored in their ability to colocalize with BMAL1 (Figure S7C). Stable expression of the constructs in synchronized cells reveals the same pattern in their ability to modulate circadian reporter expression (Figure S7D and E). Thus, the abrogation of the BMAL1–CBP binding provides a plausible mechanism whereby CHRONO might influence circadian dynamics.

### Conclusions

In summary, our data demonstrate that *Chrono* (i) oscillates with a circadian frequency in multiple tissues, (ii) physically interacts with BMAL1 and PER2, (iii) specifically reduces BMAL1/CLOCK-mediated transcription independently of CRY1, (iv) affects the free-running circadian period of mice, and (v) interferes with BMAL1–CBP binding, functionally repressing the CLOCK/BMAL1 complex and modulating the circadian acetylation of target genes. Most importantly, CHRONO knockout mice display a long free-running circadian period similar to or more drastic than six other clock components. These data establish a role for *Chrono* in the mammalian circadian oscillator. Like CIPC [18], CHRONO appears unique to the vertebrate genome. Given the repressive function of CRY proteins, the evolutionary development of additional CLOCK/BMAL1 repressors in vertebrates highlights the importance of fine control of circadian rhythms. Transcriptional oscillations can differ in their amplitude, frequency, phase, basal expression, and waveform shape. The ability to independently control these characteristics likely requires multiple, tunable genetic parameters. The specificity of CHRONO-mediated repression for BMAL1 over BMAL2, along with tissue-specific variation in the expression of BMAL1 and BMAL2, may thus facilitate local tuning of circadian oscillations.

Of course, there remain important, unanswered questions with regard to the function of CHRONO in modulating circadian dynamics. Although the abrogation of BMAL1–CBP is a plausible mechanism for CHRONO-mediated repression, it may reflect only part of its circadian function. Moreover a nuanced understanding of how this repression leads to a period-lengthening phenotype in the knockout animal will likely require a greater understanding of kinetics and network compensation. Our BiFC data demonstrate that overexpressed CHRONO co-localizes with the CLOCK/BMAL1 complex in nuclear bodies. It was previously shown that BMAL1 recruits CBP primarily when localized to promyelocytic leukemia (PML) nuclear bodies [63]. Thus the interruption of the CBP–BMAL1 binding within these nuclear structures is consistent with the potent repression induced by CHRONO overexpression (Figure 3E). Indeed, while this work was in revision, Annayev et al. [64] also reported that CLOCK/BMAL1 transcription is efficiently repressed by CHRONO(GM129). Our experimental work adds both a description of the circadian locomotor phenotype of the *Chrono* knockout mouse and an understanding of the mechanism by which this repression is mediated.

Although our work focused on the interaction between CHRONO and BMAL1, CHRONO might also influence circadian physiology through its interaction with PER2. It was recently reported that PER2 also localizes to PML nuclear bodies [65]. The importance of CHRONO/PER2 binding (Figure 3A,C), both within this complex and more generally, remain unexplored. PER2 not only binds with cryptochromes but also interacts with nuclear receptors NR1D1 and RORA [66]. Our preliminary tests (Figure S8) show that overexpression of CHRONO enhances the PER2/NR1D1 complex formation. The recruitment of this established circadian repressor provides another mechanism for CHRONO-enhanced repression of the circadian network. The importance of CHRONO/PER2 binding and a broader analysis of the role of CHRONO in the circadian network will require further study.

The extent to which CLOCK can recruit CBP/P300 independently of BMAL1 also remains unclear [67]. Given the highly redundant structure of the circadian oscillator [68], the ability of CLOCK to recruit a co-activator hints that there may be a functional paralogue of CHRONO acting on the other half of the BMAL1/CLOCK complex. Perhaps most importantly, the knockout and targeted disruption of several other clock factors have been shown to not only influence circadian period but also downstream physiological changes in metabolism [69] and sleep homeostasis [70],[71]. More detailed phenotyping of CHRONO knockout mice will be required to identify any such deficits.

Machine learning has recently been applied to complex biological problems including drug discovery [72], protein translation [73], and gene interaction networks in yeast [74]. We used a simple form of probabilistic machine learning to integrate sparse existing data whose joint distribution is hypothesized to yield a more specific ranked list of candidate genes. Although follow-up experimentation is an important part of this process, the identification of *Chrono* reflects the ability of this approach to find genes regulating circadian behavior. To our knowledge, this is the first application of these methods to identify genes responsible for complex neurological behaviors. We anticipate that the investigation of other candidates will advance the understanding of circadian rhythms. Indeed, in addition to CHRONO, our initial screening of the top 25 novel candidates identified two other proteins that both bind clock components and modulate *in vitro* circadian oscillations. To facilitate the experimental characterization of these and other candidates, a more exhaustive candidate ranking is provided in Table S2. As bona fide clock components are discovered and high-quality datasets become available, exemplar distributions can be re-evaluated and feature metrics can be improved. Thus, this integrated computational and experimental approach presents a path for leveraging genome scale data to develop insight into circadian biology.

## Materials and Methods

### Ethics Statement

All animal experiments were performed with the approval of the Institutional Animal Care and Use Committee (IACUC Protocol Numbers 801906 and 803945).

### Informatics

Unless otherwise specified, all computations were done in the R programming environment [75].

### Metric Function Construction

#### Cycling

Time-course datasets spanning 48 h with a 2-h sampling frequency obtained from pituitary, liver, and NIH 3T3 cells [25] were separately normalized using the GCRMA function (bioconductor package) [76]. The R implementation of JTK_cycle [77] was applied to each tissue-specific dataset, and the *p* value describing the probability of observing the given data under the null hypothesis of nonperiodic behavior was obtained. The cycling metric was computed from the product of the three *p* values: −log(*p*_Liver_×*p*_Pituitary_×*p*_NIH 3T3_). Thus, the cycling metric does not simply assign a gene as “cycling” or “noncycling” but provides a continuous measure reflecting the robustness of cycling in several tissues.

#### Circadian influence

Screen methods and initial processing were presented previously [28]. In brief, each gene was targeted by two distinct pools of siRNA constructs. Two replicate wells were utilized for each siRNA pool. Kinetic luminescence readings were fit sinusoidal waves to obtain an amplitude and period for each well. The log ratio between target and control circadian parameters was provided by the study authors [28]. Separate log ratios were computed for period and amplitude parameters. For each gene, the siRNA pool that induced the greatest magnitude in log change was used for further analysis. The *z* scores for the induced amplitude (*Z* *_Amp_*) and period (*Z* *_Period_*) changes, in comparison to all other targeted genes, were computed. The circadian influence metric was computed as [*Abs*(*Z* *_Period_*)+*Abs*(*Z* *_Amp_*)].

#### Interaction

The supplementary table providing the fully connected genetic interaction network was obtained from [29]. For each gene, the number of interactions with the exemplar clock list was tabulated. Only nonself interactions are included.

#### Ubiquity

Tissue ubiquity scores, which equal the total number of distinct murine tissues in which ESTs for a gene had been identified, were obtained from the authors of [30] and used as the ubiquity metric. Unlike the other features, a single cutoff value was used to discriminate the likelihood that a given gene might be a core circadian component. The cutoff was determined receiver operator curve analysis, selecting the point on the curve with maximal distance from the line of identity [78].

#### Homologene

The set of all gene groups in the Homologene database (Build 66) that have mouse and human homologues was used to represent mammalian genes [31]. For each of these Homologene groups, we looked to see if a *Drosophila melanogaster* homologue was identified.

#### Identifier mapping

Gene identifiers used from the various component datasets were all mapped onto Homologene identifiers using the flat file from the Homologene database (Build 66) [31]. Identifiers that were not listed in the Homologene database were submitted to the NCBI biological database network for mapping to the appropriate Homologene identifier [79]. Data associated with gene identifiers that remained unmapped after both attempts were ignored for further analysis.

### Evidence Factor Derivation

The derivation of circadian evidence factors closely follows that for Bayes factors [32], and our strategy follows the Naïve Bayes Classifier approach of “learning” the feature distributions from the training data. We considered an individual feature described by metric 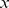, and a single arbitrary gene with observed metric value 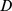. The event space is divided in two disjoint events: 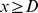 and 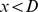. These events correspond to a randomly selected gene having a metric value at least as extreme as 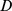 or the randomly selected gene having a metric value less than 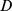. The events are labeled 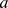 and 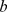, respectively. The use of an interval rather than a point allows us to regularize the sparse empirical data for the estimation. Each gene is assumed to belong to either the set of clock genes (*Cgene*) or the set of nonclock genes (*NCgene*).

By Bayes' Theorem:
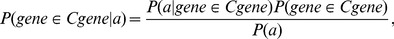
(E1)and
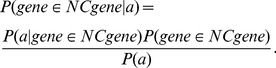
(E2)


Dividing (E1) by (E2) yields:
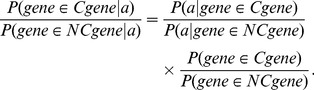
(E3)


Substituting the definition of 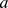, the middle term of (E3) becomes:
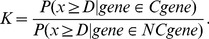
(E4)


The left-hand side of (E3) is the posterior odds of a gene being a core clock component conditional on observing a metric value greater than or equal to 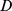. The last term represents the general odds of clock gene membership without additional feature information. Thus, the posterior odds of a gene belonging to the set of clock genes (given a metric value greater than or equal to 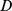) is equal to the product of 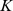 and the *a priori* odds.

### Combined Evidence

Our analysis included *n* = 5 clock gene features. For each metric 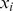, the event space is divided into two disjoint events—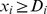 and 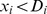 for some 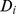
—and these events are labeled 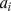 and 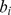, respectively. Following the steps above:
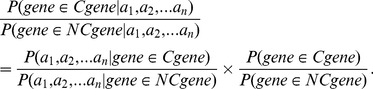
(E5)


The middle term in equation (E5) is the factor by which the *a priori* odds of clock gene membership must be adjusted to recover the posterior odds after all of the observed data. It represents the combined evidence factor (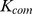) given all five features. Given the number of features, the training set of circadian clock components is too sparse to approximate the required joint distribution without some regularizing assumption. We follow the typical Naïve Bayes approach and show that, given conditional independence of the included features, 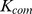 is simply the product of the individual evidence factors.

By definition, random variables 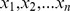 with probability density functions 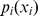 and joint probability density function 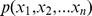 are conditionally independent given a random variable 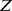 if and only if:
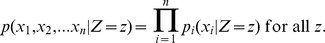



Using this definition and the definition of the events 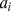, the denominator of 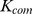 can be simplified:
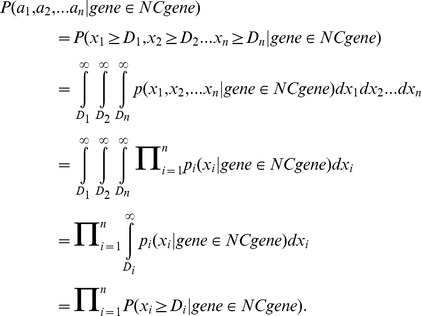



Similarly, the numerator of 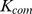 becomes 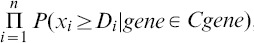, and the cumulative evidence is equal to:
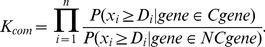
(E6)


### Computation of Evidence Factors

Given either the distribution of metric values among exemplar clock genes or the distribution among the genome at large, the probabilities of obtaining a metric greater than, or equal to, that observed was approximated with the ecdf() function in R. For any given gene and feature, the ratio of these probabilities was computed to obtain the value of 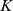. Metric values greater than the maximum value observed among exemplar clock components were assigned the same evidence factor as that maximum value. Combined evidence factors are the product of the feature-specific factors. If no data were available for a given gene and feature, this feature was ignored by setting the corresponding evidence factor to be 1. The ubiquity and homology metrics were both Boolean variables, and the standard Bayes factor formula was used for these features.

### Cross-Validation and Method Comparison

The use of combined evidence factors was compared with two prepackaged, supervised machine learning algorithms in the R programming environment: a Gaussian/Normal Naïve Bayes classifier within the “e1071” package [80] and a Flexible Naïve Bayes classifier [39] within the “klaR” package [81]. Probabilistic learning algorithms were preferred as they do not require a prior weighting of the importance of the various features [24]. For training, genes not in the exemplar clock group were labeled as nonclock genes, and the classifier was trained on the entire dataset. Genes were rank ordered on the posterior probability of clock gene membership after the model was applied to the data. For the Flexible Naïve Bayes implementation, kernel density estimation was performed with the default value for the “window parameter.” This default uses a heuristic formula to adjust the window of kernel density estimate based on the number of data points.

We sequentially removed all possible pairs of clock components from the exemplar distribution and retrained the various learning algorithms on the reduced exemplar sets, testing our ability to theoretically recover these known clock genes using different ranking cutoffs (Figure S2A). The three methods all had comparable performance using cutoffs less than ∼1,000, but the evidence factor method outperformed the other two beyond this point. The top candidates from all three methods show a very high degree of overlap (Figure S2B). We estimated the false discovery rate (FDR) of the Evidence Factor approach by combining the sensitivity analysis with an assumed total number of clock components to generate an expected number of true and false positives at different ranking thresholds (Figure S2C).

### Supporting Microarray Results in Mutant Animals and in Response to Light

Preprocessed microarray data obtained from WT and *Clock* mutant animals as reported by Miller et al was downloaded from the Circa database [47] and replotted. A single apparent outlier from the SCN data (Mutant, original time point 46) is excluded from the plot as this value was greater than any other SCN expression value from WT or mutant animals, and ∼3× the replicate measure. Cel files from the *Cry1/Cry2* double mutant were obtained from NIH GEO and normalized via GCRMA [76].

Exon-array cel files describing the transcriptional response of WT and melanopsin knockout animals to sham control and following a light pulse [57] were downloaded from NIH GEO. Data were extracted, annotated, quantile normalized, and log transformed at the gene level using the Affymetrix Expression Console package (v1.1). The probeset corresponding to *Gm129* was then separately analyzed. In both WT and knockout animals, when compared to sham control, *Chrono* expression did not significantly change 30, 60, or 120 min after light pulse.

### Experimental Confirmation of *Gm129* Cycling in Tissue Samples

#### Tissue collection

Six-week-old male C57BL/6J mice (Jackson) were housed in light-tight boxes and entrained to a 12-h light, 12-h dark schedule for 1 wk before being switched to constant darkness. Starting at circadian time (CT) 18, 2–3 mice were sacrificed per time point. Liver, white fat, and skeletal muscle samples were excised and snap-frozen in liquid nitrogen.

#### qPCR

We homogenized 2 mm^3^ tissue samples in 500 µl Trizol (Invitrogen) using a TissueLyzer (Qiagen), and total RNA was purified using RNEasy columns according to the manufacturer's protocol (Qiagen). (Reverse transcription and qPCR were carried out as per Baggs et al. [68].)

### *In Vitro* Function and Binding Experiments

#### cDNA and shRNA expression plasmids

Construction of plasmids expressing wild-type and CRY-insensitive mutant *Bmal1* and *CLOCK*, and wild-type *Cry1*, *Cry2*, and *NPAS2* cDNAs were published previously [49]. *CHRONO/C1Orf51*, *BMAL2*, and MGC library cDNAs in Sport6 vector (Invitrogen) were obtained from Open Biosystems (Huntsville, AL). *Per1*, *Per2*, and *Per3* cDNAs were published elsewhere [5].

Mammalian 2-hybrid constructs, two-hybrid reporter plasmid, Epitope-tagged cDNAs, and S-tagged CHRONO constructs were cloned using standard recombinant genetic techniques.

Hybrid *Bmal1* and *Bmal2* genes were generated by gene splicing by overlap extension (SOE) [82] with 50–60 bp primers that overlap the junctions of the *Bmal1/Bmal2* fusions. All *Bmal1/Bmal2* hybrid fusions were sequenced to verify that the full-length, in-frame fusion was generated. The pGL3P–*Per1* [17] and pGL3Basic–*Bmal1* [7] reporters are described elsewhere. The pGIPZ nonsilencing shRNAmir control and hChrono-directed shRNAs #1 (Oligo ID V2LHS_17058) and #2 (Oligo ID V2LHS_17062) constructs were purchased from Open Biosystems.

#### Transient transfections and cell-based reporter assays

Ninety-six–well Per1 promoter-luciferase reporter assays in HEK 293T cells were performed as reported elsewhere [49] with modification. We cotransfected 5 mg of a Renilla luciferase (Rluc) expression plasmid to normalize reporter activity for transfection efficiency. We used 50 mg of pGIPZ vector in shRNA cotransfections. For mammalian two-hybrid assays, 25 ng of pGL4P–4XUAS, 5 ng Rluc, 50 ng pACT, and 50 ng pBIND plasmids were transfected into HEK 293T cells in 96-well plates as previously described [49]. Transfected cells were analyzed after 24 h incubation for luciferase reporter activity with DualGlo luciferin reagent (Promega).

#### Stable transgenic cell line creation

*Per2*–*dLuc* U2OS cells were stably transfected with pcDNA3.1 vectors expressing S-tagged CHRONO wild-type and truncation mutants. The cells were grown with the treatment of selection marker (G418; Invitrogen) for 4 wk. After selection, co-IP and kinetic luminometry were performed as described.

#### Native co-IPs and Western blotting

Native co-IPs and Western blotting of epitope-tagged proteins expressed in HEK 293T cells were performed as previously described [49]. We transfected 3 µg of total plasmid DNA per each 10 cm Petri dish with 1–1.5 µg of individual pCMV-Sport6 expression plasmids transfected in each condition. For co-IP of Flag-tagged CLOCK or BMAL1 with Myc-CHRONO, 1 µg of empty pCMV-Sport vector was transfected to normalize transfections with 3 µg total DNA.

#### Isolation and quantification of RNA levels by real-time PCR

HEK 293T cells in 24-well plates at 80% confluence were transfected with 100 ng pCMV–*Bmal1* or *Bmal2*, 250 ng pCMV–*CLOCK*, and 100 ng of empty, *Cry1*, or *CHRONO* expression plasmids and FugeneHD (3 µl FugeneHD:1 µg plasmid DNA). RNA was harvested 24 h after transfection RNA levels were measured by real-time PCR as already described.

#### IP analysis for BMAL1 binding

HEK293 cells were transfected with plasmids encoding Flag–BMAL1, Flag–CLOCK, and S-tagged wild-type and mutant CHRONO constructs as indicated in the figures. At 48 h post-transfection, the cell lists were harvested in radioimmunoprecipitation assay (RIPA) buffer supplemented with a protease inhibitor cocktail (Roche) and centrifuged at maximum speed for 20 min at 4°C. Equal amounts of total protein were incubated with 2 µg of anti–S-Tag (Novagen) antibody overnight and then to a protein G-Sepharose bead slurry. The final immune complexes were analyzed by immunoblotting. Immunoblot analyses were performed on 6% or 8% sodium dodecyl sulfate polyacrylamide gels and transferred to polyvinylidene difluoride membranes (Immobilon P; Millipore). Target proteins were detected with anti–S-Tag (Novagen) and anti-Flag M2 (Sigma) antibodies. The immune complexes were visualized with HRP-conjugated secondary antibodies and ECL detection (Pierce).

#### IP analysis for PER2/NR1D1 binding

HEK 293T cells were transfected with plasmids encoding PER2–Venus, NR1D1–Flag, and CHRONO-S as indicated in the figures. At 48 h post-transfection, the cells were harvested for IP procedures as described above. Immune complexes precipitated after the overnight incubation of the cell lysates with 4 µg anti-GFP antibody (Sigma, G1544). Complexes were immunoblotted using anti-GFP, anti–S-Tag (Novagen), and anti-Flag M2 (Sigma) antibodies.

#### Chromatin IP

Both control U2OS cells and those stably overexpressing CHRONO were used in ChIP analysis. Lysates were obtained 24 and 36 h after dexamethasone synchronization. Experimental procedures to prepare chromatin were performed as described by Schmidt et al. [83]. The precleared chromatin was immunoprecipitated overnight at 4°C by agitating with 5 µg of anti–acetyl-histone H3 (Lys9) antibody (07-352, EMD Millipore). The cell extracts without incubation of antibody were used for input control. Immune complexes were collected by incubation with protein-G–coated magnetic beads (10004D, Life Technologies) and the final eluted DNA was extracted by phenol-chloroform-isoamyl alcohol (25∶24∶1) and ethanol precipitation. The primer sets used for ChIP qPCR analysis of human *Per1* promoter region spanning canonical (CACGTG) were as follows: forward primer, 5′-TCTCCCTCTCTCCTCCCTTCC-3′; reverse primer, 5′-GCCTGATTGGCTAGTGGTCTT-3′.

### BiFC and Immunofluorescence (IF) Assays

C- and N-terminal regions of an enhanced variant YFP called Venus were fused with identified constructs. Expression vectors of S-tagged full-length CHRONO (1–385), and its various deletion mutants were cotransfected with GFP–BMAL1 expression vector or BiFC fusion plasmids encoding VC–BMAL1, CLOCK–VN, or CBP–VN. At 16 h post-transfection, the cells were fixed with 4% paraformaldehyde in PBS and incubated with anti–S-tag (Bethyl Laboratories, Inc.) and anti-hC1orf51 (Santa Cruz Biotechnology) antibodies, followed by secondary antibodies conjugated to Alexa Fluor 568 (Invitrogen). Cells were visualized using fluorescein isothiocyanate and tetramethylrhodamine isothiocyanate filters in fluorescence microscopy.

### Genotype and Circadian Phenotype for Chrono Knockout Mice

#### Generation of mice containing Chrono^flx/flx^ alleles

The *Gm129* (*Chrono*) mouse strain (*Gm129* ^tm1a(KOMP)Wtsi^) was created from embryonic stem cell clone EPD0378_5_B03 generated by the Wellcome Trust Sanger Institute and made into mice by the KOMP Repository and the Mouse Biology Program at the University of California, Davis. Heterozygous mice (*Chrono^flx/+^*) on a C57BL/6 background were bred to generate homozygous (*Chrono^flx/flx^*), WT (*Chrono* ^+/+^), and heterozygous (*Chrono^flx/+^*) mice.

#### Circadian behavioral analysis

Mice were housed in individual cages within a temperature- and humidity-controlled, light-tight enclosure. Each cage contained a running wheel. Food and water were allowed *ad libitum*. Wild-type (*n* = 5), *Chrono^flx^* ^/+^ (*n* = 8), and *Chrono^flx/flx^* mice (*n* = 6) were entrained to a 12∶12 h L∶D cycle for ≥2 wk before being released into constant darkness. Locomotor activity monitoring, actogram creation, and period calculations were performed using ClockLab Data Collection (Actimetrics). Statistical analysis of period change was done through application of both a *t* test and an alternative, nonparametric Mann–Whitney test using the t.test() and wilcox.test() functions in R. Both tests resulted in a significantly (*p*≤.05) greater period among mutant as compared to wild-type mice.

### Phase Response to Light Pulse

A modified Aschoff type II procedure was used, facilitating the exposure of animals to light pulses before their free-running rhythms had drifted apart significantly [84],[85]. Animals were entrained to a 12∶12 L∶D cycle and then placed in constant darkness (D∶D) prior to a 30-min light pulse. The light pulses were initiated at zeitgeber times (ZTs) 16 or 22 on the second day of D∶D. Animals remained in DD for 7 d following the light pulse.

Daily activity onset times were determined using ClockLab Data Collection software (Actimetrics) and were exported for further analysis. The phase response was calculated as the difference between activity onset predictions as determined by prepulse and postpulse regression lines computed in R. The prepulse regression line was fit from activity onset data for 5 d prior to the light pulse. The postpulse regression line was determined from the first through seventh days in D∶D following the pulse [85].

### ShRNA-Mediated Knockdown and Kinetic Luminescence

#### Cell culture

NIH 3T3 mouse fibroblasts were cultured in DMEM supplemented with 10% fetal bovine serum and antibiotics, and grown to confluence prior to bioluminescence recording or harvesting for mRNA time courses.

#### Lentivirus

Lentiviral particles were produced by transient transfection in HEK 293T cells using the calcium-phosphate method as previously described [86]. Infectious lentiviruses were harvested at 48 h post-transfection and used to infect NIH 3T3 cells. NIH 3T3 cells were first infected with pLV7–P(Bmal1)–dLuc reporter followed by blasticidin selection to generate 3T3 reporter cells [27].

#### shRNA

Seven shRNAs targeting different regions of *Chrono* gene were designed. A nonspecific (NS) shRNA construct was used as a control. Synthetic oligonucleotides were annealed and cloned into pENTR/U6 (Invitrogen) and subsequently cloned into the pLL3.7GW vector as previously described [27]. The NIH 3T3 reporter cells were then infected with shRNA viruses.

#### Western

A fragment of *Chrono* opening reading frame (nts 352–1128) was first cloned into p3xFlag–CMV-14 vector and cotransfected with pLL3.7GW–shRNA into NIH 3T3 cells. shRNA knockdown efficiency was determined by Western blot analysis.

Primers used for cloning were as follows: forward primer, GAATTCccaccatggaactccaagggttcatacggcccctca (EcoRI); reverse primer, TCTAGAgggctgaggatccggagcaactgg (XbaI).

#### Cell harvest and qPCR

Total RNAs from NIH 3T3 cells were first prepared using Trizol reagents (Invitrogen) followed by further purification using RNeasy mini kit (Qiagen). Reverse transcription and qPCR were performed as previously described [27] except that probe and primers for *Chrono* were purchased from ABI. Transcript levels for each gene were normalized to *Gapdh*. Average relative expression ratios for each gene were expressed as a percentage of the maximum ratio at peak expression.

#### Bioluminescence recording and data analysis

Bioluminescence patterns of NIH 3T3 reporter cells were monitored using a LumiCycle luminometer (Actimetrics) as previously described [27]. Raw data were plotted. The period of the resulting luminescence data was determined through the WaveClock algorithm as implemented in R [87]. The median value of the period corresponding to the “total mode” was used. Amplitude was determined by regression to a sinusoidal waveform with the established period. To assess significance of period and amplitude changes, results for the various *Chrono* shRNAs were pooled and compared to control using the nonparametric Wilcoxon sum rank test [wilcox.test() function in R]. Both the reduction in amplitude and increase in period were significant at *p*<0.05. The data were also fit to a mixed effects model using the R package “lme4” [88]. This model incorporated a fixed effect term for *Chrono* knockdown along with a nested, random effects term for the distinct shRNAs. This model explicitly accounts for the added variance resulting from the distinct shRNA constructs in a more nuanced fashion and also demonstrated a significant (*p*<.05) reduction in amplitude along with a trend (*p* = 0.08) for increasing period.

### Reverse Transcription and qPCR

We used 1 µg total RNA to generate cDNA with the High Capacity cDNA Archive Kit using the manufacturer's protocol (Applied Biosystems). qPCR reactions were performed using iTaq PCR mastermix (BioRad) in combination with gene expression assays (Applied Biosystems) on a 7800HT Taqman machine (Applied Biosystems). *Importin 8* was used as an endogenous control for all experiments.

### Recombinant Genetic Techniques

Mammalian two-hybrid constructs were generated by PCR with primers containing the flanking restriction sites that allow for in-frame cloning of the full-length ORF (not including the start ATG codon) into pACT or pBIND plasmids (Promega). The two-hybrid reporter plasmid pGL4P–4XUAS was generated by inserting 4× repeats of the Gal4 UAS binding sites into the pGL4P vector (Promega).

Epitope-tagged cDNAs were generated by PCR with primers containing the flanking restriction sites that allow for in-frame cloning of the full-length ORF (not including the start-ATG codon) into pFlag [49] or pTag3C plasmids (Stratagene).

For plasmids expressing S-tagged CHRONO (both wild-type and truncation mutants), full-length and truncated DNA fragments of the gene were amplified with upstream and downstream primers containing S-tag-encoding sequence (KETAAAKFERQHMDS) and were subcloned into pCMV Sport6 or pcDNA3.1 expression vectors (Invitrogen) using NotI and XhoI restriction enzymes.

### shRNA Construct Sequences

The shRNA construct sequences were as follows: NS shRNA, CAACAAGATGAAGAGCACC; Sh234, GACTGGAGTTGCATCCTAT; Sh235, GAGCCAGCATTGGTGTCAT; Sh236, GACTTGGTTTCCTCACATA; Sh237, GGAGAACGTTATCTAGGAA; Sh238, GGAGCCTCGTTGCCACAGT; Sh239, GAACCTTGCTGCAGGTGGA; and Sh240, GTGTCATCCTTGTCCTCCA.

Sh 238, 239, and 240 were ultimately found to be ineffective by Western and/or PCR.

### Taqman Probe Identifiers

The Taqman probe identifiers were as follows: For *Mus musculus*: *Arntl*, Mm00500226_m1; *Arntl2*, Mm00549497_m1; *Per1*, Mm00501813_m1; *Per2*, Mm00478113_m1; *Per3*, Mm00478120_m1; *Nr1d1*, Mm00520708_m1; *Chrono* (Gm129), Mm01255906_g1; *Importin 8*, Mm01255158_m1. For *Homo sapiens*: *Arntl*, Hs00154147_m1; *Arntl2*, Hs 00368068_m1; *Clock*, Hs00231857_m1; *Per1*, Hs00242988_m1; *Per2*, Hs00256144_m1; *Nr1d1*, Hs00253876_m1; *Chrono* (C1orf51), Hs00328968_m1; *Gapdh*, Hs99999905_m1.

## Supporting Information

Figure S1 (A) Ten-fold cross-validation for machine learning approach. Two of the exemplar clock components (2/17, ∼10%) were removed from the exemplar-training list and evidence factors were recomputed based on the reduced list. Genes were reranked on the posterior probability of having a core clock function. We then recorded the ranking of the known clock components that had been excluded from the training set. This procedure was repeated after sequentially withholding all 156 possible pairs of exemplar components. (Main) The fraction of the test (withheld) clock components recovered using a given ranking cutoff (labeled sensitivity) is plotted as a function of the ranking cutoff. The evidence factor approach is compared to prepackaged implementations of a Normal/Gaussian Naïve Bayesian classifier and a Flexible Naïve Bayesian classifier. (Insert) Focused view on algorithm performance using cutoff rankings bellow 400. (B) Venn/Euler Diagram showing overlap among the top 50 candidates clock components as assessed by each of the three different machine learning algorithms. (C) Estimated FDR for evidence factor approach under different assumptions of core clock network size. The number of true core clock components is assumed to be 25, 50, or 75 genes as shown. The numbers of true and false positives were estimated from the number of true clock components, test sensitivity, and cutoff number to be screened. A dashed vertical line corresponding to a screening of the top 50 candidates is shown to facilitate comparison.
(TIFF)

Figure S2 **Initial characterization of candidate genes.** Mammalian two-hybrid screening and kinetic luminescence imaging were used to select high-probability candidate genes for more detailed evaluation. (A) The top 25 novel candidate genes (not in the exemplar distribution) were screened for physical interactions with the listed subset of clock factors. When fused with the VP16 activation domain, Cystathionine Beta Synthase (CBS) and Interferon-induced Transmembrane Protein 1 (IFITM1) demonstrated binding with a >5-fold activation of the Gal4 UAS reporter over control. GM129/CHRONO was screened in the same way and bound BMAL1 and PER2 as shown in Figure 3. As compared to NS siRNA control, siRNA mediated knockdown of (B) *Cbs* and (C) *Ifitm1* altered rhythms in synchronized NIH 3T3 fibroblasts expressing a BMAL:dLUC reporter. Data shown are mean ± standard deviation of four replicates. On initial testing, other genes among the top 25 candidates demonstrated knockdown phenotype in the NIH 3T3 system or evidence of binding, but not both.
(TIFF)

Figure S3 **The effect of core circadian oscillator mutations on** ***Chrono*** **expression.** (A) Time course microarray data from Miller et al. [46] including wild-type and *Clock* mutant animals are plotted. Data shown are average of two biological replicates. In both liver and SCN, CLOCK mutation affects *Chrono* expression level and rhythmicity. (B) Time course microarray data from Vollmers et al. [48] describing hepatic transcription from WT and *Cry1/Cry2* double knockout mice under different feeding protocols. Data were downloaded from the NIH GEO repository. GCRMA-normalized probeset values describing *Per1* and *Chrono/Gm129* expression are shown.
(TIFF)

Figure S4 **The influence of CHRONO on** ***Nr1d1*** **expression in cells overexpressing wild-type BMAL1/CLOCK or CRY-resistant BMAL1/CLOCK point mutants.** The indicated plasmids were cotransfected into HEK 293T cells and Nr1d1 expression was determined by qPCR. Average activities and standard deviations were determined from independent biological triplicates.
(TIFF)

Figure S5 **Confirmation of shRNA efficacy for kinetic luminescence experiments.** (A) Protein abundance was assessed by Western blot analysis using anti-Flag antibody in NIH 3T3 cells cotransfected with shRNA and Flag-tagged cDNA. (B) The efficiency of shRNA-mediated knockdown on endogenous transcript expression was measured by qPCR.
(TIFF)

Figure S6 **Confirmation of knockout mouse genotype.** (A) Schematic representation of wild-type (+) or transgenic allele (*Chrono* ^flx^) with knockout-first-reporter tagged insertion (KOMP repository). The transgenic allele is nonfunctional by virtue of the SV40 polyadenylation sequence (pA) inserted in the vector that acts like a STOP codon. The small arrows (a and b) show the location and direction of PCR genotyping primers. (B) PCR genotyping of DNA extracted from mouse toes of WT (*Chrono* ^+/+^), heterozygous (*Chrono* ^flx/+^), and homozygous (*Chrono* ^flx/flx^) offspring. The arrows (a and b) indicate PCR products corresponding to the targeted alleles. A size marker is shown in column M. (C) qPCR analysis for *Chrono* mRNA expression in WT, heterozygous, and homozygous Chrono knockout mice with five different qPCR primer/probes.
(TIFF)

Figure S7 **Additional data for** **Figure 6** **.** (A) Western blot showing protein bands for CBP–VN and VC–BMAL1 in the absence or presence of S-Tagged CHRONO, SPORT6, or SPORT6-S. Invariant protein levels suggest that changes in complementation signal (Figure 6B and C) result from changes in protein complex formation rather than changes in CBP–VN or VC–BMAL1 abundance. (B) Only S-tagged CHRONO constructs containing the 108–285 region repress CLOCK/BMAL1-mediated Per1–Luciferase reporter activity. (C) CHRONO truncation mutants were coexpressed along with a BMAL1–GFP construct. Cellular localization was visualized via IF analysis using an S-tag antibody. Intact CHRONO and truncation mutants containing the 108–285 region colocalized with BMAL1–GFP in the nucleus. (D) Real-time bioluminescence analysis using the *Per2*:luc reporter cells (U2OS) stably expressing the indicated constructs. Data shown are the average of four independent experiments. (E) Quantitative analysis of amplitudes of oscillations shown in (D). Error bar indicates standard error of the mean.
(TIFF)

Figure S8 **The influence of CHRONO on PER2/NR1D1 complex formation.** HEK 293T cells were transfected with plasmids encoding PER2–Venus, NR1D1–GFP, and CHRONO–S as indicated in the figure. Endogenous protein was immunoprecipitated with anti-GFP (which also targets Venus) followed by immunoblotting as indicated. PER2/NR1D1 binding appears enhanced in the presence of CHRONO.
(TIFF)

Table S1 **Data matrix showing average fold-activation of the 4XUAS:Luciferase reporter (±S.D.) with specified Gal4 and VP16 fusion constructs cotransfected into HEK 293T cells.**
(DOC)

Table S2 **Excel file containing feature metrics for the top 1,000 genes as assessed by evidence factor ranks.** The ranking of these gene obtained using the Gaussian and Flexible Naïve Bayes classifiers are also reported.
(XLS)

## Abbreviations

BIFC: bi-molecular fluorescence complementation
Chrono: computationally highlighted repressor of the network oscillator
co-IP: co-immunoprecipitation
D∶D: constant darkness
ECDF: empirical cumulative distribution function
FDR: false discovery rate
GFP: green florescent protein
H3–K9: histone H3 lysine 9
HEK 293T: Human Embryonic Kidney 293 cells containing the SV40 T-Antigen
IF: immunofluorescence
L∶D: light∶dark
NIH 3T3: immortalized mouse fibroblast cell line
NS: nonspecific
PML: promyelocytic leukemia
qPCR: quantitative PCR
SCN: suprachiasmatic nuclei
U2OS: human osteosarcoma cell line
YFP: yellow florescent protein
ZT: zeitgeber time
