# Supplementary figures and images for: Machine Learning Helps Identify CHRONO as a Circadian Clock Component
Source: PLoS Biol. 2014 Apr 15;12(4):e1001840. doi: 10.1371/journal.pbio.1001840 (PMC3988006; doi:10.1371/journal.pbio.1001840)

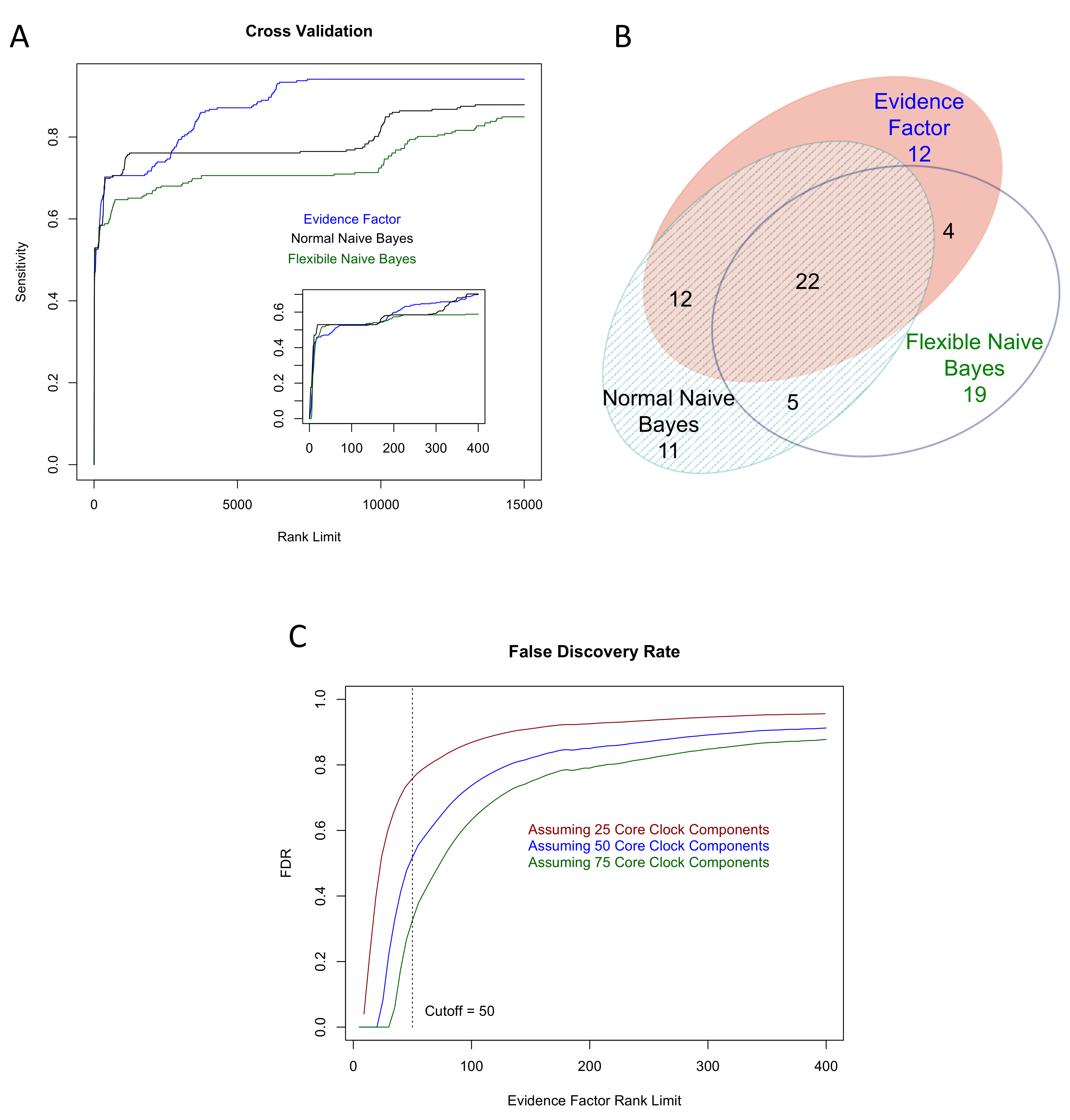

Supplement: Figure S1 — (A) Ten-fold cross-validation for machine learning approach. Two of the exemplar clock components (2/17, ∼10%) were removed from the exemplar-training list and evidence factors were recomputed based on the reduced list. Genes were reranked on the posterior probability of having a core clock function. We then recorded the ranking of the known clock components that had been excluded from the training set. This procedure was repeated after sequentially withholding all 156 possible pairs of exemplar components. (Main) The fraction of the test (withheld) clock components recovered using a given ranking cutoff (labeled sensitivity) is plotted as a function of the ranking cutoff. The evidence factor approach is compared to prepackaged implementations of a Normal/Gaussian Naïve Bayesian classifier and a Flexible Naïve Bayesian classifier. (Insert) Focused view on algorithm performance using cutoff rankings bellow 400. (B) Venn/Euler Diagram showing overlap among the top 50 candidates clock components as assessed by each of the three different machine learning algorithms. (C) Estimated FDR for evidence factor approach under different assumptions of core clock network size. The number of true core clock components is assumed to be 25, 50, or 75 genes as shown. The numbers of true and false positives were estimated from the number of true clock components, test sensitivity, and cutoff number to be screened. A dashed vertical line corresponding to a screening of the top 50 candidates is shown to facilitate comparison. (TIFF) [file pbio.1001840.s001.tif]

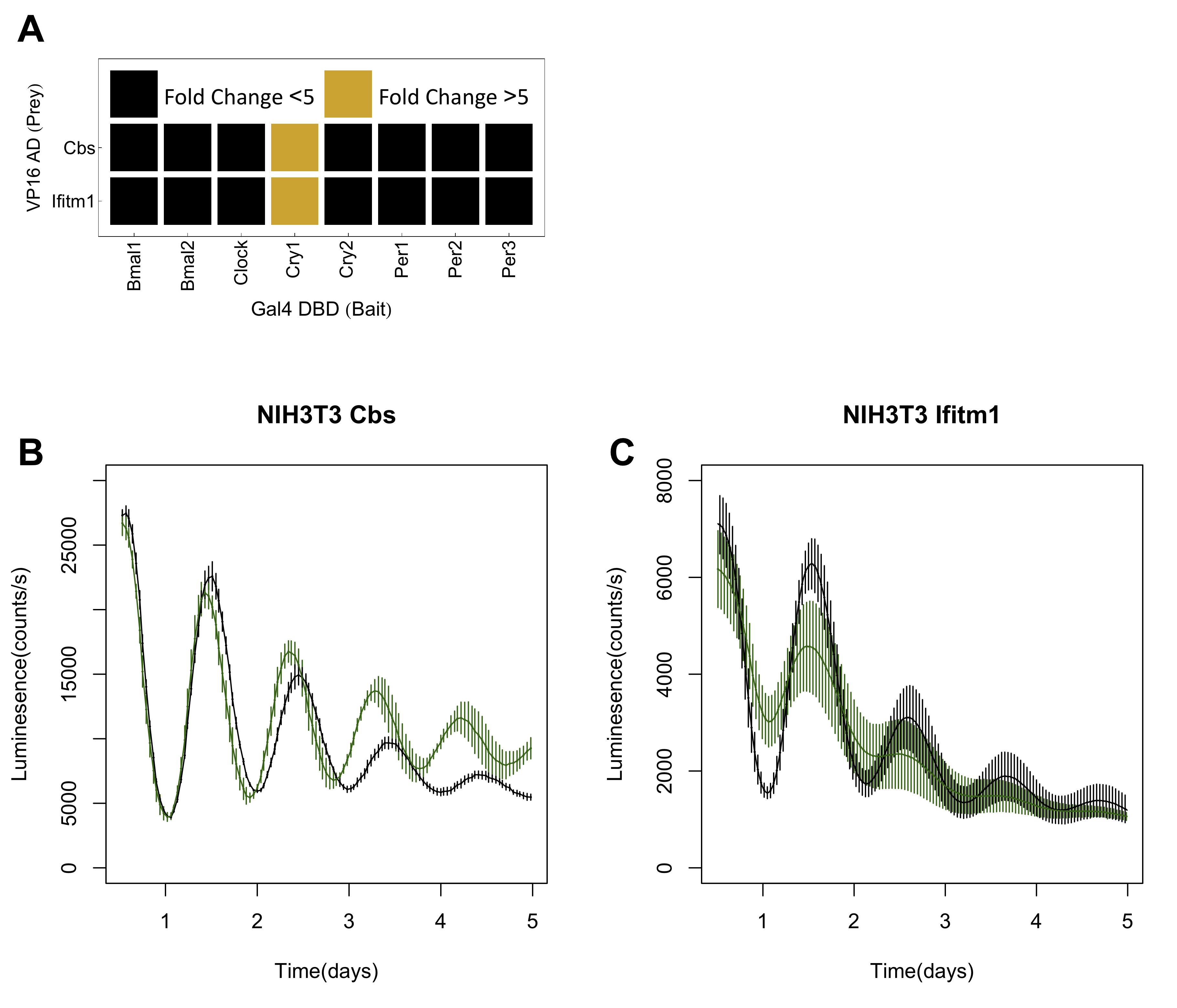

Supplement: Figure S2 — Initial characterization of candidate genes. Mammalian two-hybrid screening and kinetic luminescence imaging were used to select high-probability candidate genes for more detailed evaluation. (A) The top 25 novel candidate genes (not in the exemplar distribution) were screened for physical interactions with the listed subset of clock factors. When fused with the VP16 activation domain, Cystathionine Beta Synthase (CBS) and Interferon-induced Transmembrane Protein 1 (IFITM1) demonstrated binding with a >5-fold activation of the Gal4 UAS reporter over control. GM129/CHRONO was screened in the same way and bound BMAL1 and PER2 as shown in Figure 3. As compared to NS siRNA control, siRNA mediated knockdown of (B) Cbs and (C) Ifitm1 altered rhythms in synchronized NIH 3T3 fibroblasts expressing a BMAL:dLUC reporter. Data shown are mean ± standard deviation of four replicates. On initial testing, other genes among the top 25 candidates demonstrated knockdown phenotype in the NIH 3T3 system or evidence of binding, but not both. (TIFF) [file pbio.1001840.s002.tif]

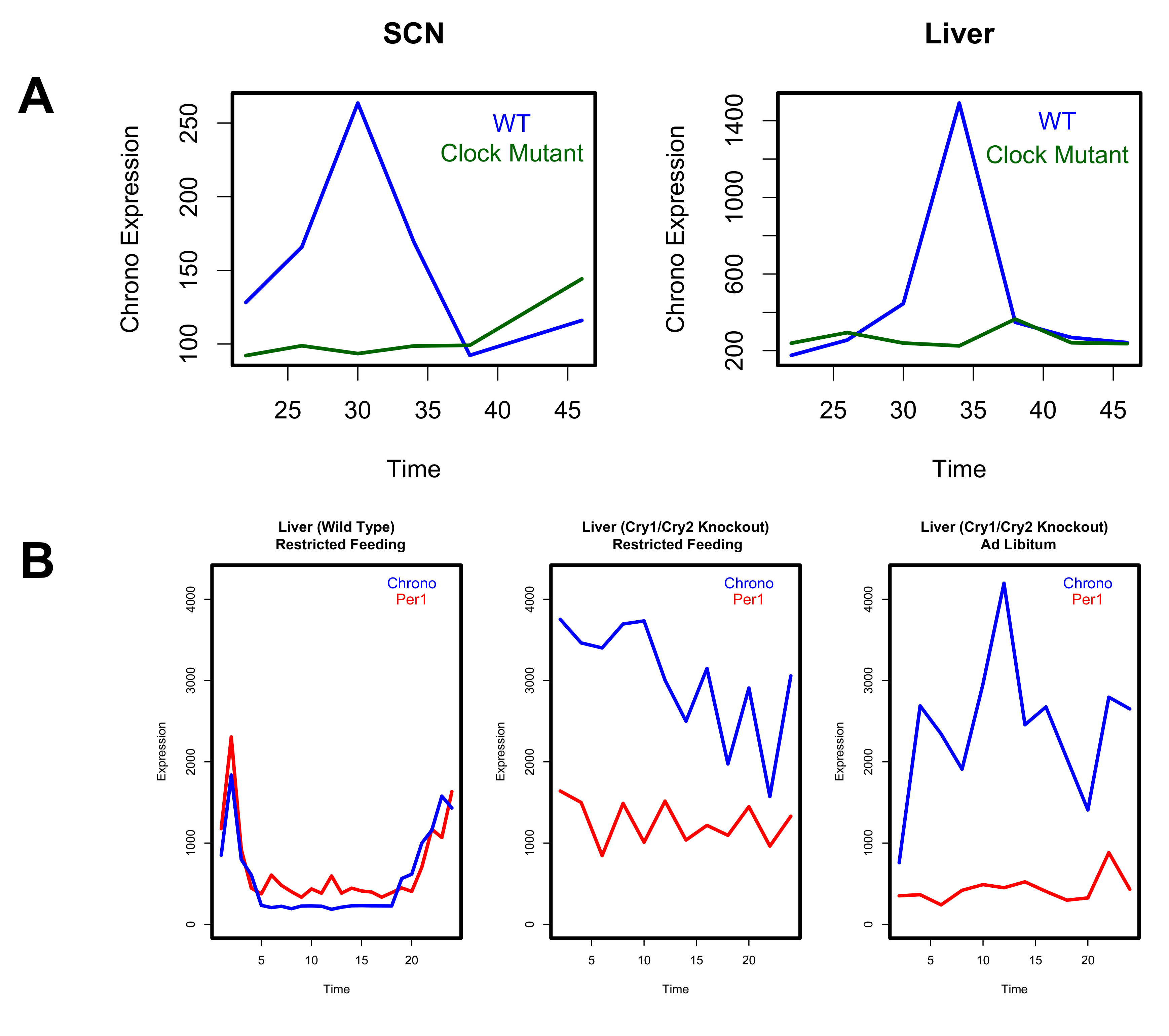

Supplement: Figure S3 — The effect of core circadian oscillator mutations on Chrono expression. (A) Time course microarray data from Miller et al. [46] including wild-type and Clock mutant animals are plotted. Data shown are average of two biological replicates. In both liver and SCN, CLOCK mutation affects Chrono expression level and rhythmicity. (B) Time course microarray data from Vollmers et al. [48] describing hepatic transcription from WT and Cry1/Cry2 double knockout mice under different feeding protocols. Data were downloaded from the NIH GEO repository. GCRMA-normalized probeset values describing Per1 and Chrono/Gm129 expression are shown. (TIFF) [file pbio.1001840.s003.tif]

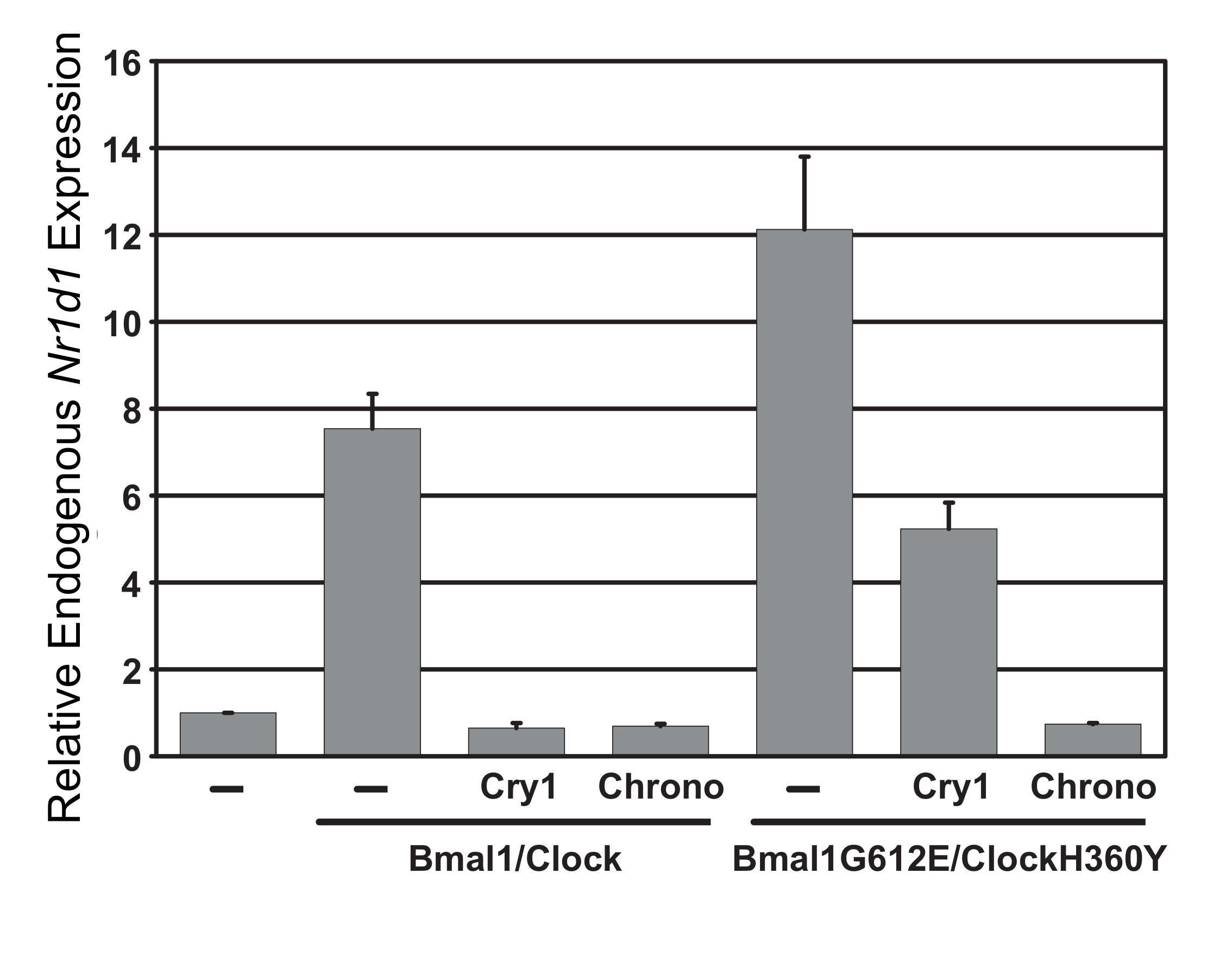

Supplement: Figure S4 — The influence of CHRONO on Nr1d1 expression in cells overexpressing wild-type BMAL1/CLOCK or CRY-resistant BMAL1/CLOCK point mutants. The indicated plasmids were cotransfected into HEK 293T cells and Nr1d1 expression was determined by qPCR. Average activities and standard deviations were determined from independent biological triplicates. (TIFF) [file pbio.1001840.s004.tif]

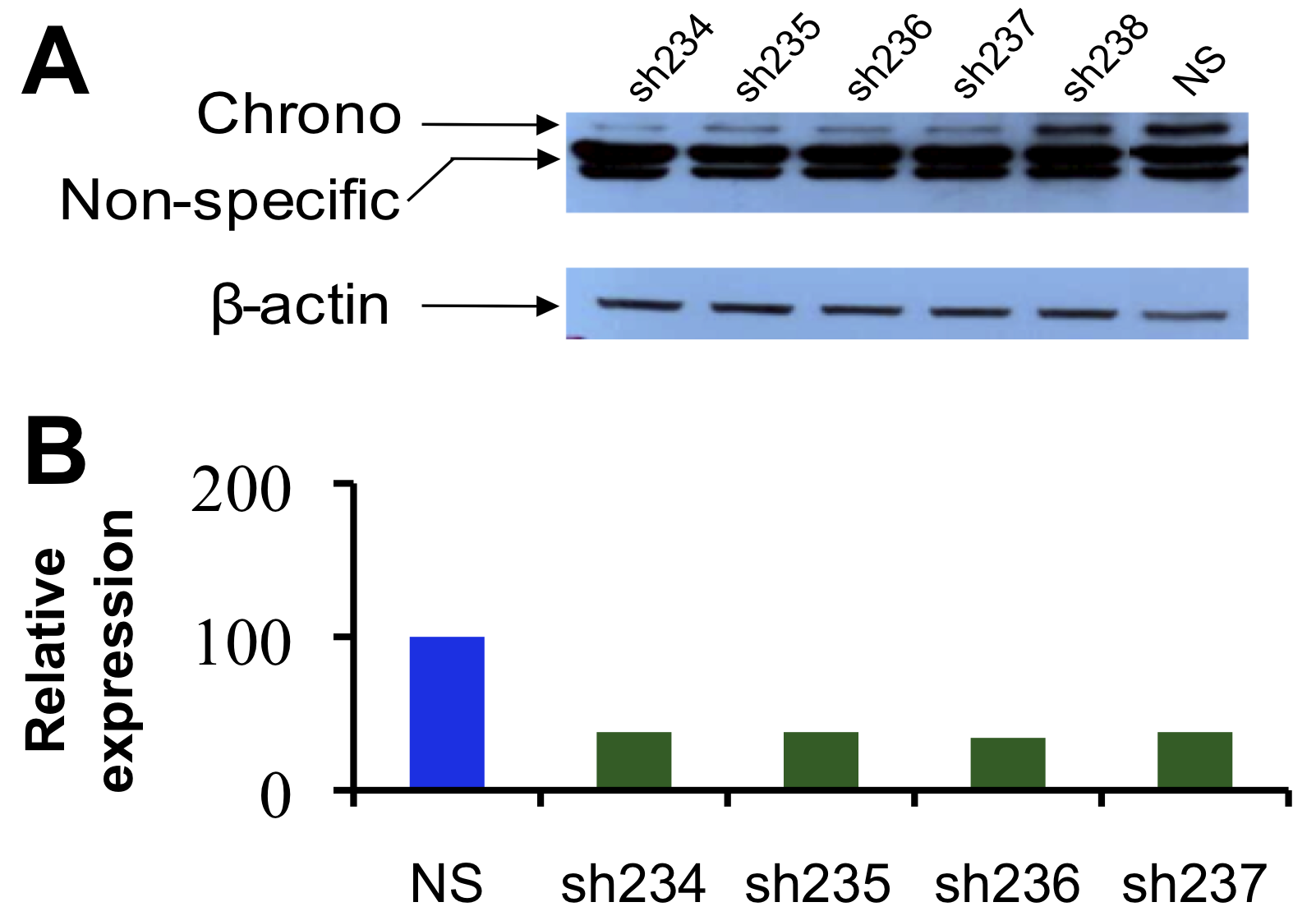

Supplement: Figure S5 — Confirmation of shRNA efficacy for kinetic luminescence experiments. (A) Protein abundance was assessed by Western blot analysis using anti-Flag antibody in NIH 3T3 cells cotransfected with shRNA and Flag-tagged cDNA. (B) The efficiency of shRNA-mediated knockdown on endogenous transcript expression was measured by qPCR. (TIFF) [file pbio.1001840.s005.tif]

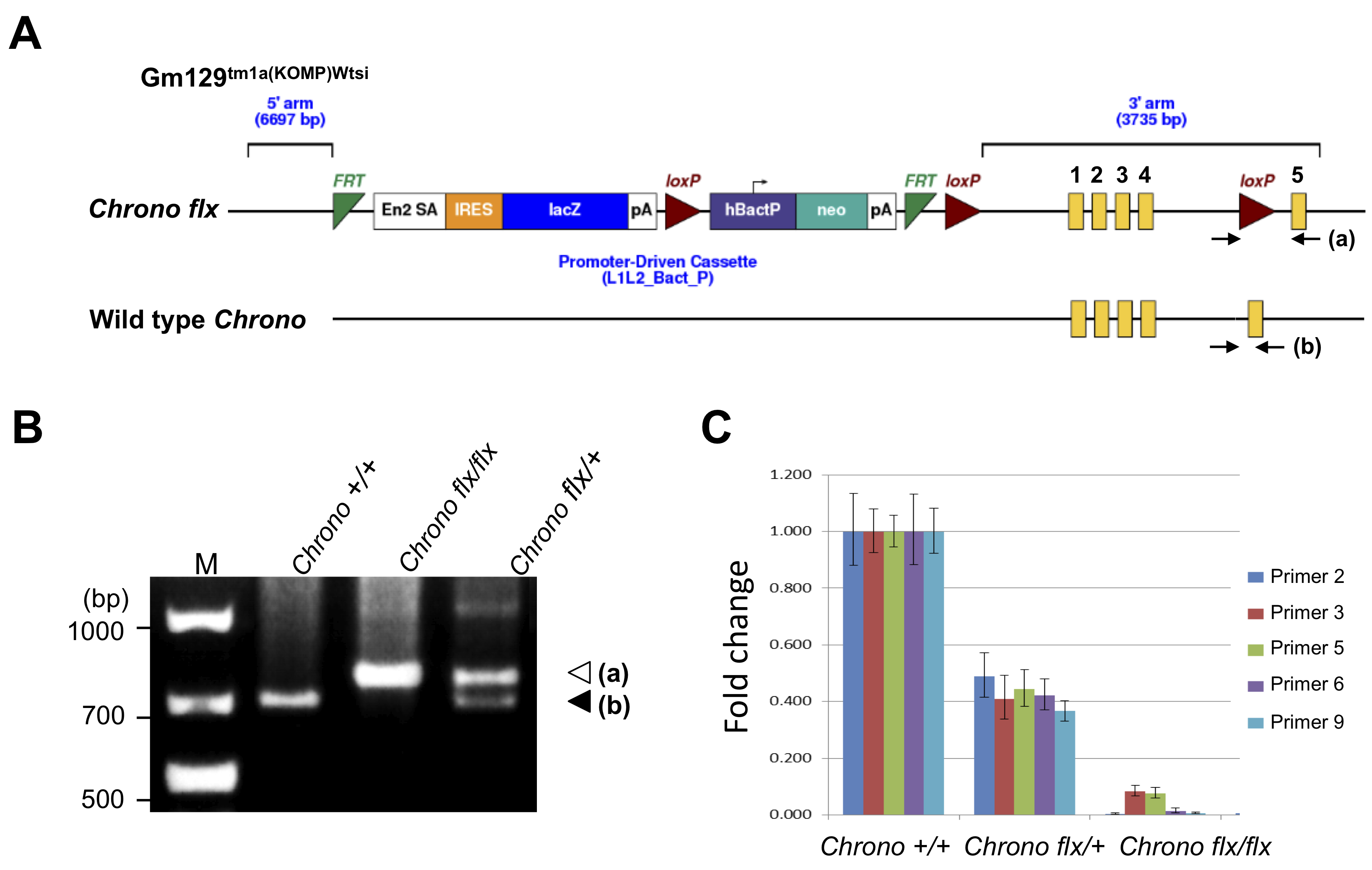

Supplement: Figure S6 — Confirmation of knockout mouse genotype. (A) Schematic representation of wild-type (+) or transgenic allele (Chrono flx) with knockout-first-reporter tagged insertion (KOMP repository). The transgenic allele is nonfunctional by virtue of the SV40 polyadenylation sequence (pA) inserted in the vector that acts like a STOP codon. The small arrows (a and b) show the location and direction of PCR genotyping primers. (B) PCR genotyping of DNA extracted from mouse toes of WT (Chrono +/+), heterozygous (Chrono flx/+), and homozygous (Chrono flx/flx) offspring. The arrows (a and b) indicate PCR products corresponding to the targeted alleles. A size marker is shown in column M. (C) qPCR analysis for Chrono mRNA expression in WT, heterozygous, and homozygous Chrono knockout mice with five different qPCR primer/probes. (TIFF) [file pbio.1001840.s006.tif]

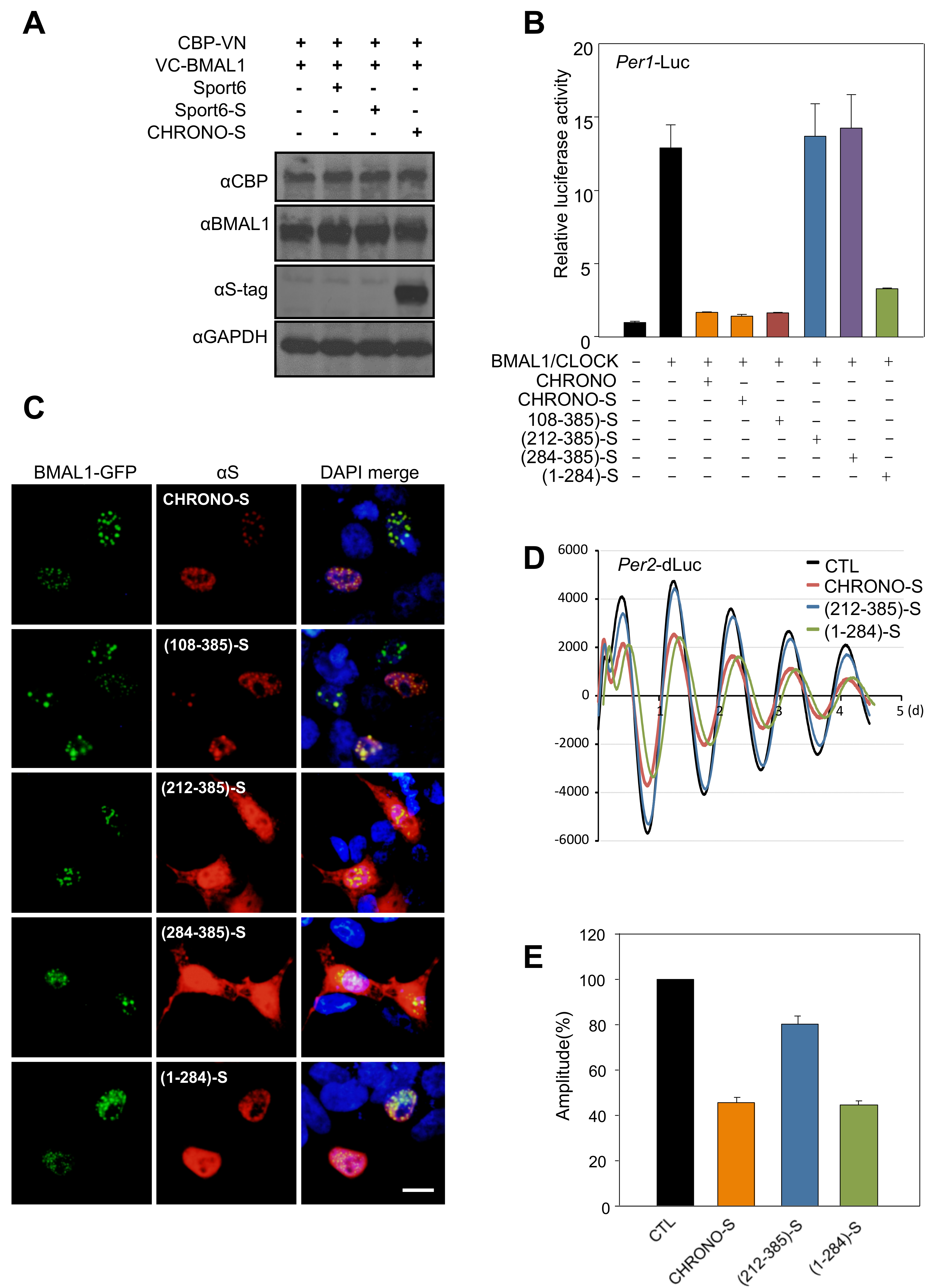

Supplement: Figure S7 — Additional data for Figure 6 . (A) Western blot showing protein bands for CBP–VN and VC–BMAL1 in the absence or presence of S-Tagged CHRONO, SPORT6, or SPORT6-S. Invariant protein levels suggest that changes in complementation signal (Figure 6B and C) result from changes in protein complex formation rather than changes in CBP–VN or VC–BMAL1 abundance. (B) Only S-tagged CHRONO constructs containing the 108–285 region repress CLOCK/BMAL1-mediated Per1–Luciferase reporter activity. (C) CHRONO truncation mutants were coexpressed along with a BMAL1–GFP construct. Cellular localization was visualized via IF analysis using an S-tag antibody. Intact CHRONO and truncation mutants containing the 108–285 region colocalized with BMAL1–GFP in the nucleus. (D) Real-time bioluminescence analysis using the Per2:luc reporter cells (U2OS) stably expressing the indicated constructs. Data shown are the average of four independent experiments. (E) Quantitative analysis of amplitudes of oscillations shown in (D). Error bar indicates standard error of the mean. (TIFF) [file pbio.1001840.s007.tif]
