# Supplementary material for: Machine Learning Helps Identify CHRONO as a Circadian Clock Component
Source: PLoS Biol. 2014 Apr 15;12(4):e1001840. doi: 10.1371/journal.pbio.1001840 (PMC3988006; doi:10.1371/journal.pbio.1001840)
Supplement: Table S1 — Data matrix showing average fold-activation of the 4XUAS:Luciferase reporter (±S.D.) with specified Gal4 and VP16 fusion constructs cotransfected into HEK 293T cells. (DOC) [file pbio.1001840.s009.doc]

|  | **Bmal2** | 1.00 ± 0.09 | 0.90 ± 0.02 | 0.91 ± 0.15 | 1.65 ± 0.09 | 0.34 ± 0.04 | 1.01 ± 0.08 | 1.45 ± 0.05 | 7.16 ± 0.72 | 5.92 ± 0.53 | 1.43 ± 0.23 | 1.67 ± 0.24 |
| --- | --- | --- | --- | --- | --- | --- | --- | --- | --- | --- | --- | --- |
|  | **Bmal1** | 1.00 ± 0.12 | 11.6 ± 0.45 | 1.14 ± 0.03 | 1.66 ± 0.02 | 1.14 ± 0.06 | 1.98 ± 0.24 | 2.59 ± 0.22 | 9.10 ± 2.22 | 1.92 ± 0.11 | 2.13 ± 0.14 | 2.35 ± 0.13 |
|  | **Npas2** | 1.00 ± 0.05 | 1.31 ± 0.06 | 0.85 ± 0.04 | 1.07 ± 0.15 | 0.45 ± 0.01 | 0.79 ± 0.02 | 0.97 ± 0.01 | 1.43 ± 0.20 | 1.12 ± 0.29 | 17.6 ± 2.67 | 298 ± 30.8 |
|  | **Clock** | 1.00 ± 0.07 | 2.96 ± 0.44 | 1.21 ± 0.05 | 6.68 ± 0.12 | 0.63 ± 0.04 | 1.66 ± 0.16 | 1.61 ± 0.17 | 10.3 ± 0.76 | 1.26 ± 0.10 | 31.6 ± 6.43 | 158 ± 5.04 |
|  | **Cry2** | 1.00 ± 0.12 | 1.83 ± 0.19 | 38.9 ± 2.66 | 85.2 ± 11.2 | 12.7 ± 1.02 | 0.66 ± 0.08 | 1.11 ± 0.08 | 60.6 ± 5.46 | 12.4 ± 0.42 | 9.38 ± 0.26 | 3.02 ± 0.27 |
| **Gal4 (Bait)** | **Cry1** | 1.00 ± 0.04 | 1.77 ± 0.06 | 57.0 ± 4.98 | 85.2 ± 11.2 | 12.7 ± 1.02 | 0.66 ± 0.08 | 1.11 ± 0.08 | 60.6 ± 5.46 | 12.4 ± 0.42 | 9.38 ± 0.26 | 3.02 ± 0.27 |
|  | **Per3** | 1.00 ± 0.07 | 4.02 ± 0.62 | 46.4 ± 5.41 | 5.37 ± 0.14 | 1.48 ± 0.11 | 1.81 ± 0.17 | 2.61 ± 0.32 | 1.66 ± 0.19 | 1.47 ± 0.28 | 1.45 ± 0.20 | 0.91 ± 0.27 |
|  | **Per2** | 1.00 ± 0.10 | 21.3 ± 5.07 | 50.0 ± 3.54 | 34.0 ± 2.82 | 4.01 ± 0.38 | 41.5 ± 1.37 | 45.7 ± 4.79 | 9.06 ± 0.55 | 0.45 ± 0.03 | 3.52 ± 0.24 | 3.52 ± 0.09 |
|  | **Per1** | 1.00 ± 0.09 | 1.74 ± 0.09 | 15.5 ± 1.27 | 40.1 ± 4.71 | 0.50 ± 0.04 | 10.3 ± 0.80 | 7.23 ± 0.16 | 1.44 ± 0.11 | 0.83 ± 0.04 | 1.41 ± 0.15 | 1.22 ± 0.01 |
|  | **Chrono** | 1.00 ± 0.04 | 2.51 ± 0.37 | 0.97 ± 0.18 | 25.5 ± 2.00 | 1.43 ± 0.25 | 0.91 ± 0.12 | 1.10 ± 0.09 | 1.82 ± 0.12 | 0.65 ± 0.05 | 27.1 ± 1.68 | 0.87 ± 0.01 |
|  | --- | 1.00 ± 0.05 | 1.11 ± 0.10 | 1.39 ± 0.26 | 2.24 ± 0.65 | 0.53 ± 0.11 | 1.49 ± 0.29 | 1.02 ± 0.18 | 2.42 ± 0.60 | 1.23 ± 0.06 | 1.29 ± 0.04 | 1.16 ± 0.10 |
|  |  | **---** | **Chrono** | **Per1** | **Per2** | **Per3** | **Cry1** | **Cry2** | **Clock** | **Npas2** | **Bmal1** | **Bmal2** |
|  |  | **VP16 fusion (Prey)** | | | | | | | | | | |
